# Cooperative foraging between dolphins and fish-eating killer whales

**DOI:** 10.1038/s41598-025-22718-4

**Published:** 2025-12-11

**Authors:** Sarah M. E. Fortune, Xi Cheng, Keith Holmes, Andrew W. Trites

**Affiliations:** 1 Department of Oceanography, Dalhousie University, Halifax, NS Canada; 2 Marine Mammal Research Unit, Institute for the Oceans and Fisheries, University of British Columbia, Vancouver, BC Canada; 3 Leibniz Institute for Zoo and Wildlife Research (IZW), Berlin, Germany; 4 Hakai Institute, Victoria, BC Canada

**Keywords:** Behavioural ecology, Community ecology

## Abstract

Interspecies associations during foraging can range from competition to cooperation but are often complex and difficult to interpret. Using aerial drone and biologgers (n=9) equipped with video, acoustic and inertial sensors, we recorded interactions between fish-eating northern resident killer whales (*Orcinus orca*) and Pacific white-sided dolphins (*Lagenorhynchus obliquidens*) in the presence of adult Chinook salmon (*Oncorhynchus tshawytscha*). We observed frequent co-occurrence and coordinated movements, with the killer whales orienting towards the dolphins and following them to depth. We also recorded reduced echolocation and rolling movements by the killer whales in the presence of dolphins, suggesting that the whales may eavesdrop on dolphin echolocations to scan broader areas to locate large Chinook salmon—prey that are too big for the dolphins to capture and swallow whole. Captured fish were brought to the surface by the killer whales and broken apart for sharing with other matrilineally related pod members—while the accompanying dolphins scavenged scraps. No antagonistic interactions or avoidance behaviours were observed between the killer whales and dolphins. The spatially and temporally coordinated foraging behaviours we recorded suggest northern resident killer whales and dolphins opportunistically engage in cooperative foraging, which may improve the ability of killer whales to detect Chinook, while dolphins benefit by scavenging prey scraps—highlighting the need for further investigation into the ecological implications of these interspecific encounters.

**Supplementary Information:**

The online version contains supplementary material available at 10.1038/s41598-025-22718-4.

## Introduction

Interactions among different species of marine mammals are frequently observed, yet the behavioural mechanisms underlying these associations remain largely speculative^1–4^. One such puzzling relationship occurs between Pacific white-sided dolphins (*Lagenorhynchus obliquidens*) and northern resident killer whales (*Orcinus orca*) along the coast of British Columbia, Canada, where these two cetacean species are often seen within meters of each other^5–7^.

Northern resident killer whales are highly social, matrilineal specialists that feed almost exclusively on large adult Chinook salmon^8,9^. In contrast, Pacific white-sided dolphins are generalists that consume a more varied diet that includes such species as herring, squid, and smaller-sized salmonids^10–12^. Despite some dietary overlap, field observations suggest that resident killer whales generally tolerate the dolphins’ presence unless outnumbered by large aggregations^6^.

The reasons why Pacific white-sided dolphins and resident killer whales associate are speculative. One possibility is kleptoparasitism, wherein the dolphins may be stealing prey from the killer whales. Another is that the dolphins are seeking protection from mammal-eating transient (Bigg’s) killer whales^6,7^ and to a lesser extent, large sharks^6^. Bigg’s killer whales utilize the same areas as northern resident killer whales but the two ecotypes generally avoid each other^13^. It is also possible that the dolphins are associating to learn acoustic signals (e.g., acoustic detection of the mammal-eating ecotype) needed to evade predation—particularly since fish-eating and mammal-eating killer whale are acoustically distinguishable^14^. Finally, the dolphins and killer whales may be engaging in cooperative foraging to increase their probabilities of capturing and consuming Chinook salmon—although the direct benefits that either receives may not be substantial.

While cooperative foraging within species is well documented among delphinids and other marine mammals^15–18^, evidence for interspecific cooperation is limited^1,19,20^. To address this gap and assess the other hypothesized reasons for Pacific white-sided dolphins and resident killer whales to associate with each other, we obtained aerial observations (drones), underwater video, passive acoustic recordings, and 3-dimensional dive behaviour (suction-cup attached CATs biologgers)^21^. Combining these datasets, we sought to characterize behavioural dynamics of the interspecific association between northern resident killer whales and dolphins and evaluate whether the outcomes of theses interspecific interactions showed evidence of competition, cooperation or another form of strategic association.

## Materials and methods

### Data collection

We combined aerial and underwater data to obtain high-resolution information about the feeding behaviours of northern resident killer whales in the north island waters of Vancouver Island, British Columbia, Canada (Johnstone Strait, Queen Charlotte Strait and Queen Charlotte Sound) between August 15 - 30, 2020. Our primary research vessel was a 16 m wooden hull, live-aboard named M/V Gikumi, which was used for focal follows and Unmanned Aerial System (UAS) operations. A smaller, 6 m aluminum research vessel called the Steller Quest was used to tag the killer whales and conduct some focal follows.

Northern resident killer whales were equipped with suction-cup attached Customizable Animal Tracking solutions tags (CATs) using an adjustable 6–8 m carbon fiber pole. Killer whales were photo-identified in the field based on their unique markings (e.g., saddle patch, dorsal fin shape and trailing edge). Only males and non-lactating females >4 years of age were selected for tagging. The multi-sensor CATs tags were programmed to continuously record high-resolution dive data from a time-depth recorder (50 Hz sampling rate). Killer whale vocalizations and feeding related sounds (i.e., echolocation clicks and prey-handling ‘crunches’)^14,22^ were continuously recorded using a 96 kHz HTI passive acoustic recorder (36 kHz upper bound recording rate) that was built into the tag. Underwater video was collected from a forward-facing camera built into the CATs tag both continuously and at set intervals (i.e., duty cycling). We duty-cycled the camera trigger to enable 24-hour deployments and programmed the tag to record video based on a light threshold that allowed up to 2 minutes of recording when minimum light levels were met. For shorter deployments, we programmed the tag to record video continuously—even when light levels were insufficient. All data was stored on the archival device and were downloaded once the suction-cups had detached from the killer whale via galvanic release, and the tag was retrieved. Tag deployment settings were chosen based on the time of day that tagging occurred (i.e., 24-hour tags were preferentially used during early morning deployments).

Focal follows of tagged killer whales and collection of unmanned aerial system (UAS; DJI Inspire 2 quadcopter) data was subsequently collected. The UAS was flown within 1 km of the research vessel, always remaining within line-of-sight. The UAS was flown at an average altitude of 45 m for a duration of 20 minutes at a time. Video and still images were recorded during flights for behavioural analysis using a Zenmuse *X5S* camera and Olympus 25 mm f1.8 lenses.

After deploying a tag, we followed the tagged animal until either the tag detached from the whale (e.g., short deployments of 6–8 hours), or until sunset for longer deployments (>9 hours). Focal follows were conducted from either the M/V Gikumi or the Steller Quest depending on weather and sea conditions. During the follows, the tagged animal’s location (latitude, longitude, distance and bearing), behaviour, group size and cohesion were continuously recorded (approximately every 2 minutes). Boat-based still images of the killer whale’s dorsal fin and right side of the saddle patch were taken using a Nikon SLR camera for photo-identification. The behaviour of the killer whale immediately after tagging (which included minor reactions such as a flinch, and moderate reactions such as barrel rolls) and the tag location and orientation on the body were also photo-documented.

The suction cups temporarily attached the tags to the killer whales and were released via a galvanic mechanism, which was pre-corroded based on the desired tag attachment time (6–24 hrs). However, the exact time of release depended on environmental conditions (e.g., water temperature and salinity) as well as the dive behaviour of the tagged killer whale (i.e., deep diving to colder bottom water delays corrosion, while extended periods in the warmer surface waters accelerates corrosion) and the physical interactions between individuals and bottom substrate. Wildlife Computers SPOT 363 A and 363 C satellite-telemetry tags provided satellite locations to initiate the search for the released tag, and an ARGOS goniometer and UHF directional antenna and receiver were used to determine the bearing of the floating tag, relative to the vessel.

Our data was collected in accordance with Animal Research: Reporting of *In* Vivo Experiments (ARRIVE). All killer whale data (e.g., drone, biologging and focal follow) were obtained with institutional approval from The University of British Columbia (Animal Care Permit no. A19-0053) and Fisheries and Oceans Canada (Marine Mammal Scientific License for Whale Research no. XMMS 6 2019), and all methods were performed in accordance with the relevant guidelines and regulations.

### Behavioural analysis

Detailed movement tracks of tagged killer whales were recorded during focal follows. While following our tagged killer whale (from 100–200 m) using visual and UHF signal detections (yagi antenna and receiver) we obtained detailed information about each killer whale’s horizontal movement. Using the GPS location of our vessel position^23,24^ and the bearing and distance of the killer whale, we calculated the actual position of the killer whale using the ‘destPoint’ function in the ‘geosphere’ program in R (i.e., killer whale tracks). To better understand the foraging ecology of killer whales and the depths at which the killer whales were hunting and catching their prey, we georeferenced the killer whale-tracks with bathymetric data from the British Columbia 3 Arc-Second Bathymetric Digital Elevation model generated by NOAA’s National Geophysical Data Center and National Centers for Environmental Information using QGIS.

To quantitatively assess the behavioural interactions of the killer whales and Pacific white- sided dolphins, and test hypotheses about the applicability of the cooperative or competitive framework, we used an open-source, multiplatform program (BORIS – Behavioural Observation Research Interactive Software)^25^ to calculate time-budgets for specific activities for multiple subjects (e.g., tagged killer whales and associated dolphins). Using BORIS and a pre-defined (Table S1) set of point behaviours (those that occur at one specific time only) and state behaviours (those that occur overtime and have an associated start and end time), we calculated the duration, occurrence, and intervals of behavioural events of interest. We quantified the behaviour for the entire tag attachment period for four killer whales that had confirmed interactions with dolphins (as seen from vessel-based observations). We also identified a set of foraging, social, travel and resting related behaviours based on behavioural observations made from the video and hydrophone-recorded passive acoustics.

Several studies have demonstrated that resident killer whales strongly rely on echolocation clicks and buzzes for foraging^26–29^, which we also used to identify prey searching behaviour. However, previous studies were not able to directly measure foraging activity and instead relied on acoustic data to infer feeding from kinematic biologging data^26–29^. The underwater CATs camera allowed us to directly observe feeding behaviour (hunting, successful capture, prey handling and sharing), providing a new perspective and a more complete dataset for analyzing interspecies interactions that was not available in other published biologging studies. Since echolocation clicks could also be used in non-foraging contexts, we used underwater camera footage to confirm there were congruent foraging behaviours accompanying the detected foraging- related echolocation clicks (e.g., dive depth, tortuosity, rolling, increased flow noise, and visual confirmation of salmon prey)^27^. Prey captures were confirmed both auditorily by hearing low- frequency ‘crunches’^26,27,29^ and visually by seeing a fish in the killer whale’s mouth and/or seeing clouds of blood, scales and tissue concurrent with a head jerk.

### Dive behaviour analysis

We used ‘diveMove’ in R^30^ to correct the time-depth recorder data using the zero-offset method, after downsampling to 1 Hz. Based on a minimum dive threshold of 3 m (50% of the body length of a 6 m adult resident killer whale), we used ‘diveMove’ to determine the phases (descent, bottom, ascent) and compute summary dive statistics (dive duration, maximum depth, surface recovery time, etc.).

To gain understanding of the vertical dimension of the killer whale and dolphin interactions, we matched dive data timestamps with the ethogram data derived from BORIS using underwater video and audio recordings. In particular, we were interested in knowing the depth the killer whale was at when: 1) dolphins were present in the video; 2) dolphins were near the head of the killer whale; 3) killer whales and/or dolphins were echolocating; 4) Chinook salmon kills were made; 5) prey handling ‘crunches’ were acoustically detected; 6) scales were observed while the killer whale consumed the fish; and 7) prey sharing occurred between killer whales. We then extracted the associated depth measurement from the zero-offset corrected dive data for 7 dolphin-associated and foraging-related behaviours.

Tri-axial inertial sensing data (accelerometer, magnetometer and gyroscope) were calibrated and initially processed using a MATLAB tools package^21^. Briefly, this involved correcting the tag frame to match the killer whale frame, identifying tag slips and calculating the orientation, motion and position for each tag deployment—otherwise referred to as pitch, roll and heading (PRH) data. PRH data were further processed in R to calculate: 1) circular variance in heading which is a measure of the degree of change in swim heading (radians); 2) deviation in peak jerk (m s^−3^) which is the standard deviation of the rate of change of acceleration during peak jerk; 3) roll at peak jerk (degrees) which represents x-axis acceleration changes that coincide temporally with peak jerk; and 4) vectorized dynamic body acceleration (VeDBA) which is the sum of total dynamic and static acceleration from the x, y and z axis with static acceleration (i.e., gravity) removed. VeDBA provides a measure of mechanical work^31^.

### Statistical analysis of behaviours

To quantitatively test whether killer whale behaviours were significantly different during sympatric associations, we calculated the total minutes of video where behaviours of interest (killer whale associations, echolocation, resting, kelp interactions and vocalizations) occurred for periods when dolphins were present and absent. We also calculated the duration of time individuals engaged in these behaviours and the proportion of the total video time this represented. A Fisher’s exact test was used to evaluate the significance of the association between the minutes of video a particular behaviour was observed during two time periods—one where dolphins were present and the other when dolphins were absent.

To investigate whether there were impacts on killer whale dive behaviour from when dolphins were absent or present, we used linear-mixed effects models with the nlme package^32,33^ in R version 4.0.3^34^. Based on the visual detection of dolphins from the underwater camera, we used dolphins present or absent as a fixed-effect, and used maximum depth (m), dive duration (secs), bottom time (secs) and bottom distance (m) as the response variables. We used likelihood ratio tests to determine how the presence or absence of dolphins affected killer whale dive behaviour. The Akaike’s information criterion (AIC) was used to indicate model support, and the model with the lowest AIC value was deemed to be the ‘best’ model. To account for temporal autocorrelation, we used a continuous autoregressive process within day (i.e., CAR(1) process)^9^. We tested model assumptions using graphs of standardized residuals and log and square-root transformed our response variables to ensure normality.

### Acoustic analysis

All acoustic data from the HTI passive acoustic recorder were manually examined for the presence of foraging related acoustic behaviours by generating spectrograms using the sound analysis software Raven Pro 1.6 (Center for Conservation Bioacoustics; sampling rate=96 kHz, FFT-size=512 or 1024, 85% overlap, Hanning window). During the analysis, we simultaneously listened to the recordings and viewed the spectrograms and periodically referred to the underwater footage (when available). Depending on the complexity and frequency of the sounds of the target species, the recordings were played back at reduced speed (0.25 to 0.5 original speed). Spectral peak mean values from Pacific white-sided dolphin clicks are 22.2, 26.6, and 37.3 kHz^35^, which we used to identify Pacific white-sided dolphin clicks. Killer whale echolocation clicks by comparison had lower frequency peaks between 12 and 19 kHz^36^.

Following previous methodolog^37^, consecutive echolocation clicks from the target animal (tagged killer whale or associated dolphins) were assigned to the same click train if they were separated by inter-click intervals (ICI, seconds) of ≤2s. Furthermore, killer whale and Pacific white-sided dolphin echolocation clicks with an ICI >100 ms were classified as slow clicks, while click trains were defined as a series of three or more clicks with an ICI > 10ms and < 100ms, and buzzes had clicks with an ICI ≤10 ms. Separating echolocation clicks into groups based on ICI was important because regularly spaced clicks are produced during prey searching, with an increasing repetition rate at an approach phase, while buzzes are an integral part of prey capture, enabling precise target localization^38^.

We used a combination of acoustic cues, such as the presence of characteristic low- frequency components with relatively stable amplitudes^39^ to identify which sounds were likely produced by the tagged killer whale, instead of by nearby conspecifics. Since the tags used in our study only had one hydrophone (CATs), localization was not possible because we could not calculate the time difference of arrival through triangulation as is commonly done for biologgers with multiple hydrophones (DTAGs)^40^. However, we analysed underwater videos to confirm there were no conspecifics associated with the tagged killer whales within a body length during the detected echolocation events. We also used the video to record dolphin associations with the tagged killer whales. However, the CATs camera (forward facing) only provided information about dolphin presence near the head of the tagged whale. Consistent with the method described in the behavioural analysis, the tagged killer whales that emitted the detected echolocation clicks also displayed congruent foraging behaviours in underwater camera footage. We similarly used characteristic broadband dolphin clicks that had stable amplitudes to identify which sounds were produced by Pacific white-sided dolphins associated with the tagged killer whales, and which were produced by dolphins at a distance.

By examining the scrolling display of the spectrograms and waveforms both visually and aurally in Raven Pro 1.6, we also investigated the times when both killer whales and dolphins produced echolocation clicks, and the type of their respective echolocation clicks (i.e., clicks, click trains, fast click train to buzzes)^26–29^ to understand their acoustic interactions. We visualized the temporal acoustic occurrence of Pacific white-sided dolphins and killer whales using spectrograms. Additionally, we marked the crunching sounds and the punching sounds heard when killer whales captured their prey. Crunching sounds are considered indicative of prey processing^27,29^, while punching sounds are produced when killer whales come into contact with the prey with force and speed.

To determine the acoustic interactions between killer whales and dolphins during associations, we compared the start and end times of the echolocation events of both species and identified the concurrent echolocations and the alternating echolocating patterns. We also calculated the overall duration of killer whale echolocations with and without dolphins present to further examine how killer whales may benefit from cooperative hunting with dolphins (Tables S1 & S2).

## Results

**Figure 1:**
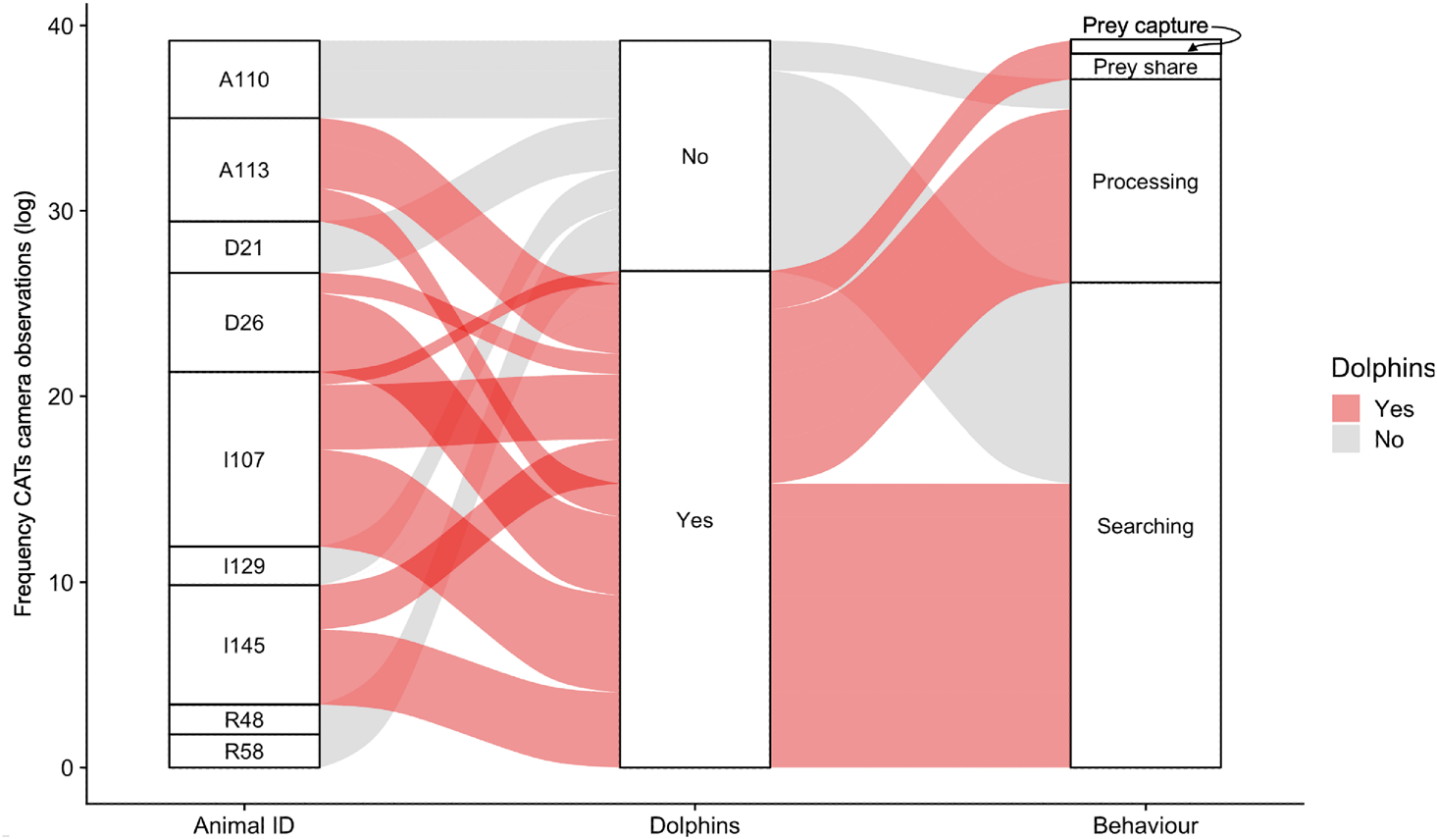
Alluvial plot showing the log transformed frequency of behavioural events logged from underwater CATS camera recordings for all tagged (Animal ID) northern resident (n=7) and southern resident (n=2) killer whales. Whether or not each killer whale was documented on the underwater video recordings interacting with dolphins ( or ) is indicated along with their engagement in foraging related behaviours (kill, prey capture, prey share (as receiver or giver), processing and searching).

**Figure 2:**
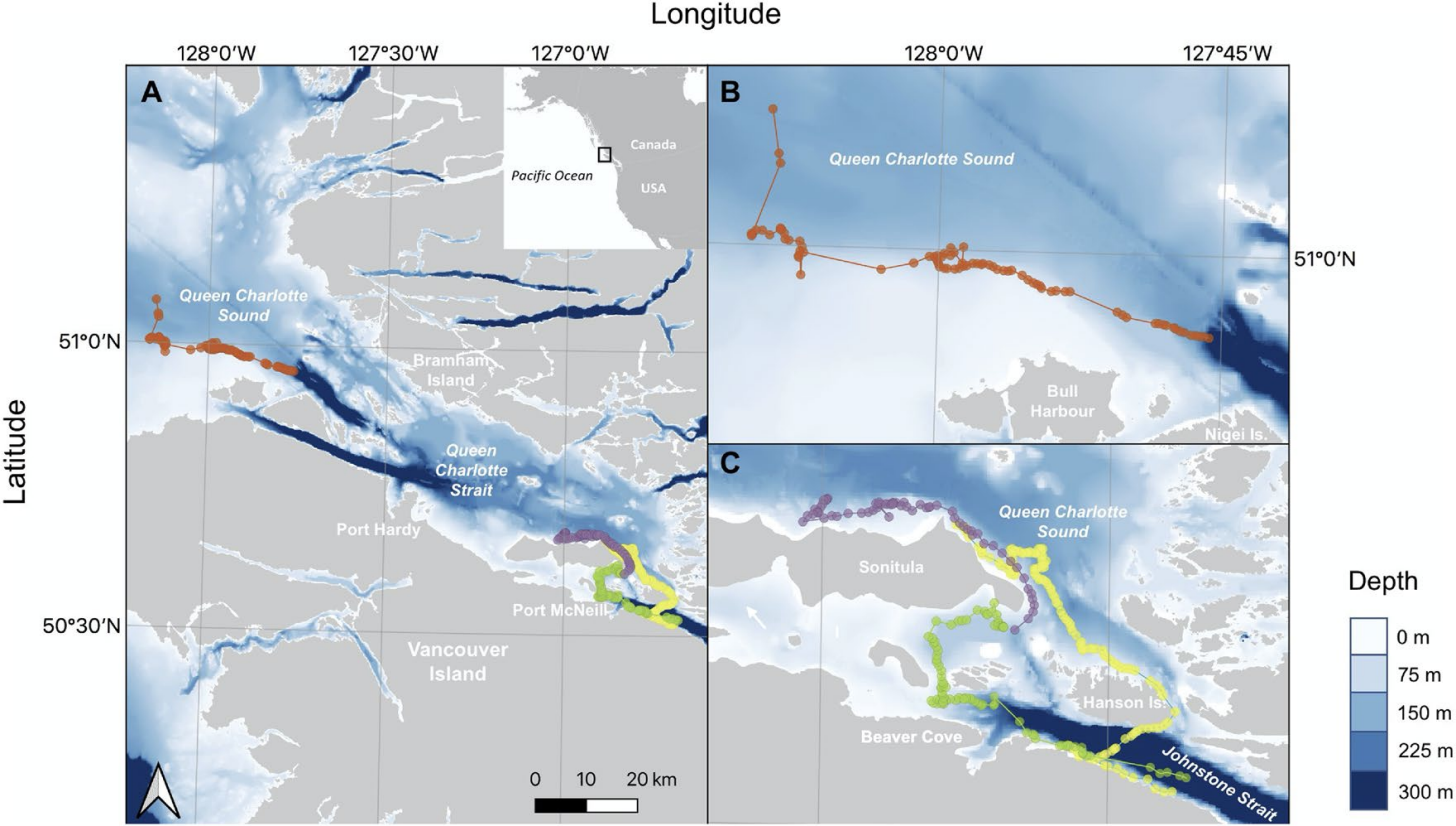
Tracks of four northern resident killer whales with dolphin associations ( = A113, = I145, = D26, = I107) tagged in the North Island Waters (British Columbia, Canada) during August 2020. Spatial movement for remaining five killer whales that did not interact with dolphins was the same as those that interacted with dolphins with the exception of no observations in Queen Charlotte Sound. Maps show bathymetric data (**A**), and zoomed in movements for animals A113, I145 and D2 (**B**) and I107 (**C**) based on killer whale location obtained during vessel based focal follows. NOAA bathymetric data were linearly interpolated based on 5 depth bins. Maps generated using QGIS 2.18 https://qgis.org/en/site/forusers/download.html.

Tagged killer whales belonged to different matrilines and foraged in the North Island Waters of Vancouver Island (British Columbia, Canada) (Figs. 1 & 2). Analysis of underwater video recordings obtained from CATs tags provided visual evidence of opportunistic association between four northern resident killer whales (n=9 tagged; Fig. 1) and numerous Pacific white-sided dolphins (IDs A113, I107, I145, D26; Table 1) over four days. There were 258 unique events of dolphins traveling near the head of tagged killer whales (n=149 belonging to I145, n=88 for D26, n=14 for A113 and n=7 for I107), resulting in a cumulative total of 63 minutes for I145, 50 mins for D26, 110 mins for I107 and 18 mins for A113. Tag durations were between 5.2 and 14.0 h (Table 1). All killer whales that were recorded interacting with dolphins engaged in foraging related behaviours that included successful salmon kills (observed catching and killing fish), prey captures (observed with fish in mouth), prey sharing, processing food (acoustic crunches, clouds of blood, pieces of fish), and searching (echolocating, frequent direction changes and acceleration in speed). Simultaneous increases in heading variance and increased swimming speed were also observed during periods of killer whale echolocation.

**Table 1:** Summary information for the four tagged killer whales (Animal ID) with dolphin interactions.

**Animal ID**	**Sex**	**Age**	**Matriline**	**Date**	**Start**	**Stop**	**Duration (hrs)**	**Camera**	**% Duration of association (hrs)**
A113	F	4	A54	8/22/2020	8:00	16:00	8.00	Intermittent	1%
I107	M	16	I17	8/25/2020	10:00	23:59	13.98	Intermittent	2%
I145	?	6	I65	8/30/2020	10:45	15:59	5.23	Continuous	21%
D26	?	10	D11	8/31/2020	10:09	19:00	8.85	Continuous	12%

All animals were from different matrilines^41^ and two were of known sex (one female and one male; F and M) and two were of unknown (?) sex. Animals A113 and I145 were juveniles, and I107 and D26 were adults. The times the tag was attached (Start) and detached (Stop) from the killer whale are provided in PDT and the total duration of tag attachments is provided in hours (Duration). Whether the CATs camera (Camera) was programmed to duty-cycled (intermittent) or continuously record (continuous) is also indicated—with ‘% duration of association (hrs)’ reflecting the cumulative amount of time dolphins were observed near the head of each tagged whale.

**Figure 3:**
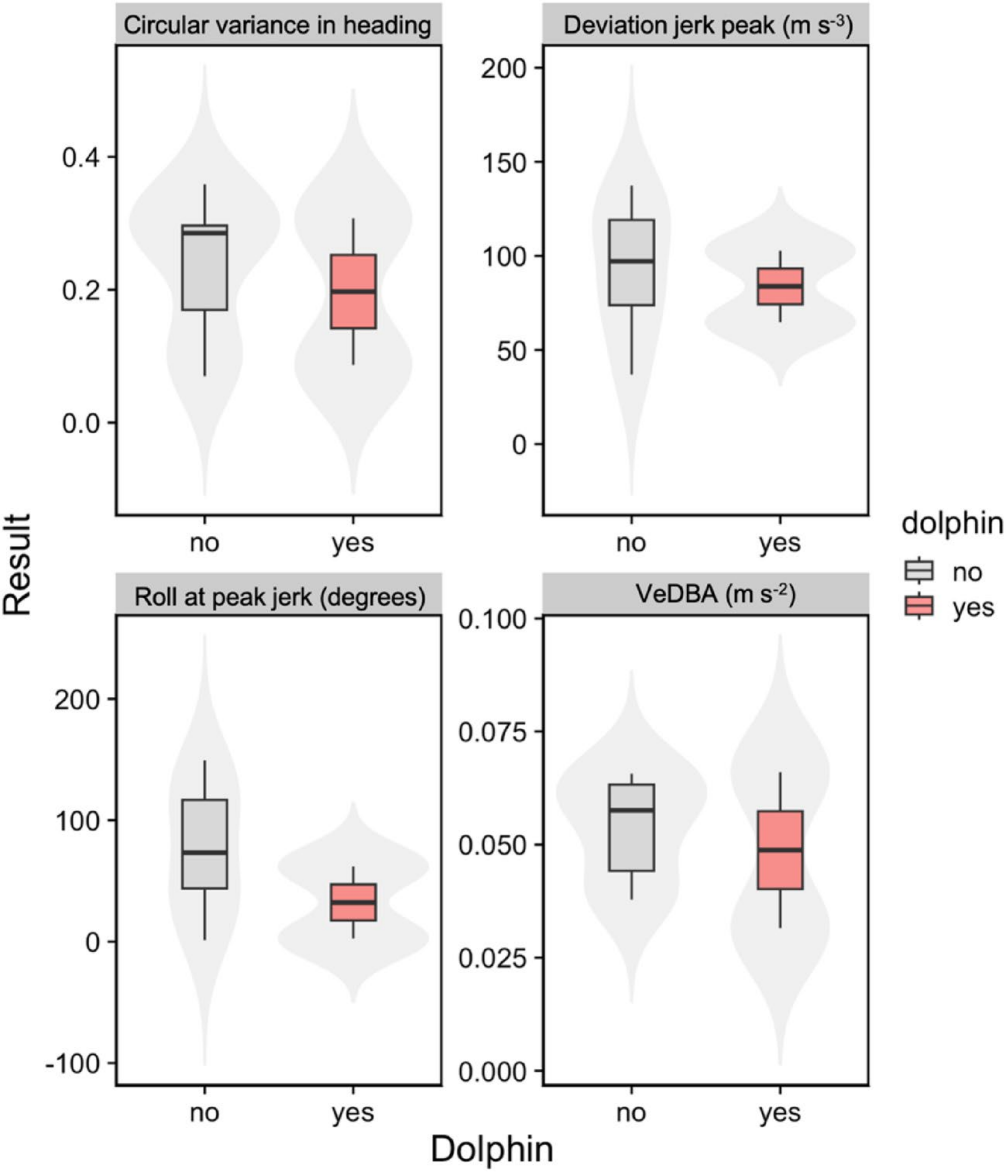
Tri-axial accelerometer, magnetometer and gyroscope data from the bottom phase of foraging dives (n=32) for three individuals that made successful prey captures while associated (: D26, I145 and I107) and unassociated (: I107) with dolphins. Circular heading variance provides a measure of the degree of change in swim heading (radians). Deviation in peak jerk (m s^−3^) reflects the standard deviation of the rate of change of acceleration during peak jerk. Roll at peak jerk (degrees) reflects x-axis acceleration changes that coincide temporally with peak jerk. VeDBA (m s^−2^) is the vectorized dynamic body acceleration (sum of total dynamic and static acceleration from the x, y and z axis with static acceleration (gravity) removed) and provides a measure of mechanical work^42^.

**Figure 4:**
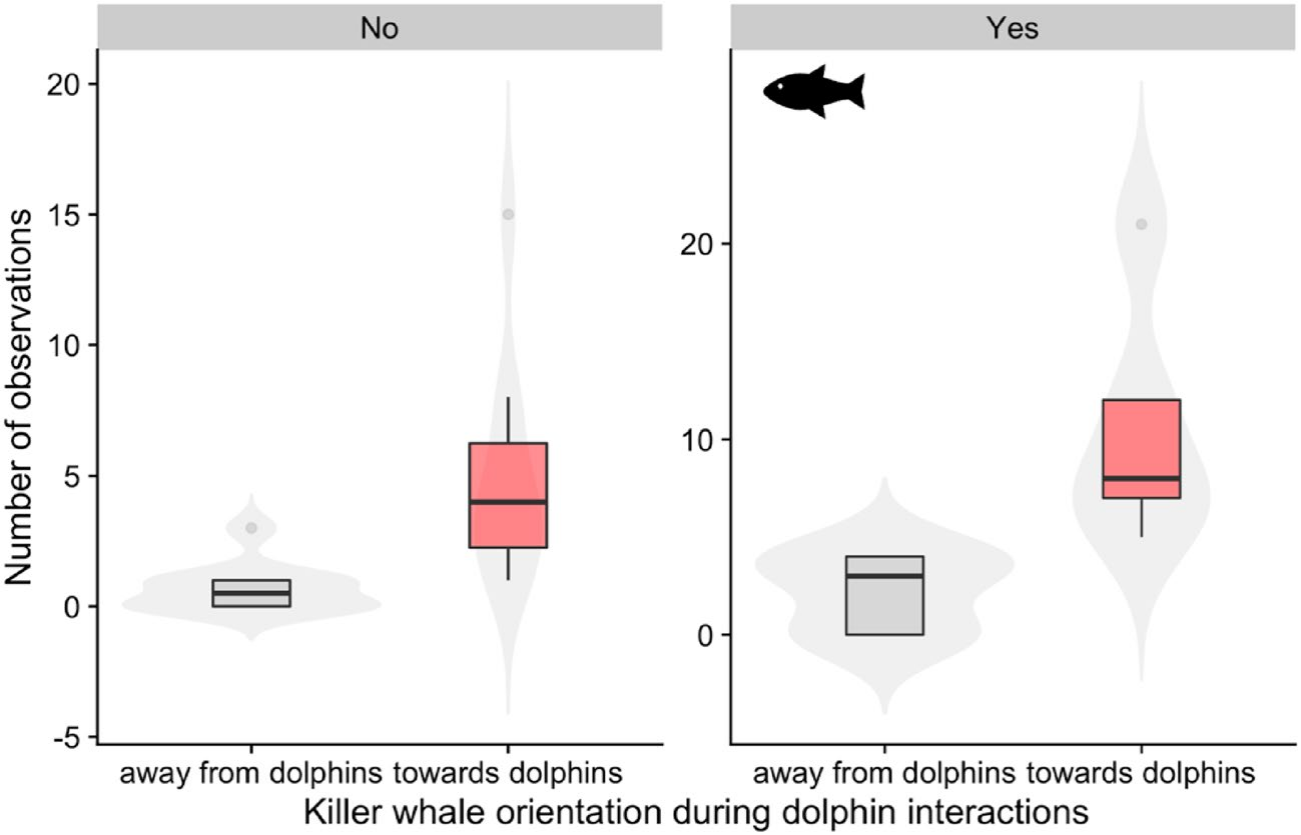
Drone observations (n=120) of killer whale orientation during dolphin interactions during events when fish were successfully captured (yes) or not (no). Instances when killer whales made movements towards dolphins are indicated with  and movements away are in .  shaded violin plots indicate the distribution of the data. Horizontal black lines in the boxplots indicate the median.  points represent outliers.

**Figure 5:**
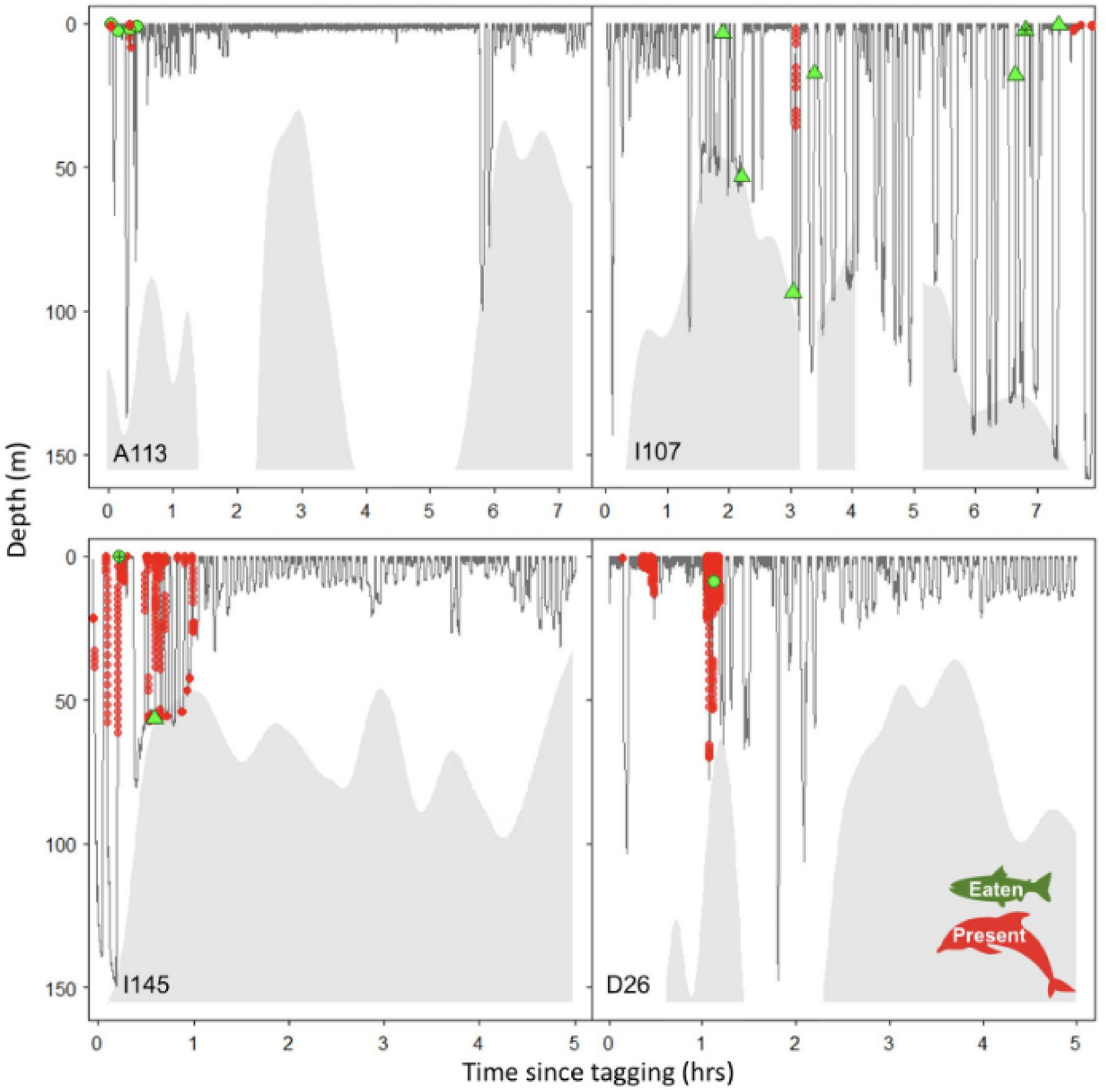
Diving depths over 5–8 h intervals for 4 northern resident killer whales (A113, I107, I145 and D26). Data points indicate when: 1) dolphins were recorded near the head of a tagged killer whale ( circles); 2) fish was shared by another killer whale (circle ; + indicates dolphin scavenging shared fish); 3) tagged whale caught a fish (triangle ; * indicates dolphin scavenging prey capture). Bathymetric data associated with known killer whale locations ascertained during focal follows (shaded in ). Total numbers of fish consumed were 4 for killer whale A113, 7 for I107, 2 for I145 and 1 for D26.

**Figure 6:**
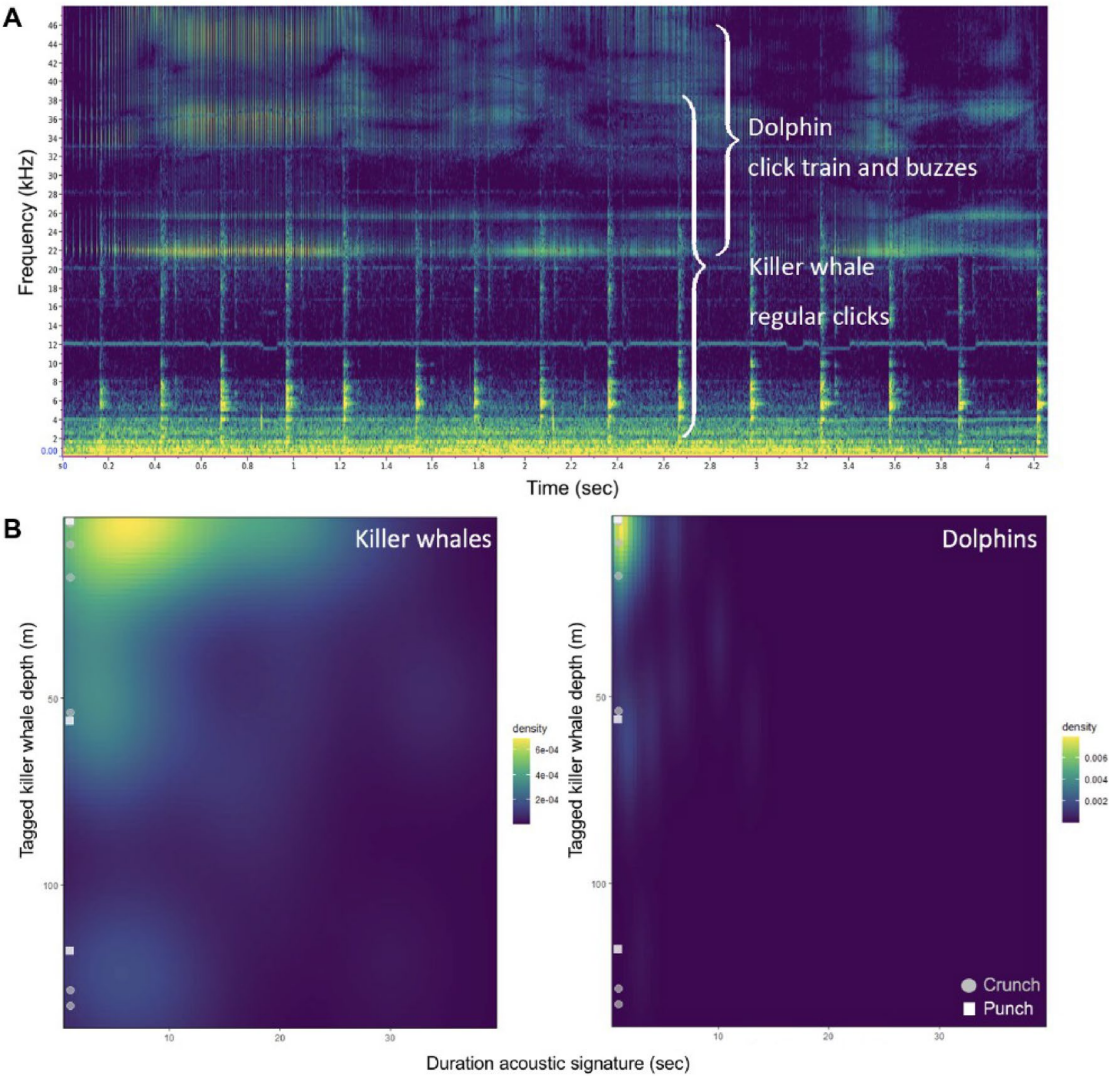
(**A**) Spectrogram of the temporal acoustic co-occurrence of Pacific white-sided dolphin (top) and killer whale (bottom) echolocation clicks (sampling rate= 96kHz, FFT- size=512, 85% overlap, Hanning window). (**B**) heatmap of depth (m) of detection of acoustic events (clicks, click trains and buzzes) by the duration of the acoustic detection (sec) for killer whales and dolphins, with z-axis indicating the density of detected acoustic events (brighter colour indicates higher density of acoustic detections). Overlaid are prey handling sounds—crunches (grey circle) and punches (white square)—for tagged killer whales. Overall, both species echolocated throughout the water column with the highest densities occurring in shallow waters, followed mid-water echolocations at 50 m. Only killer whales were observed echolocating at depths exceeding 100 m.

**Figure 7:**
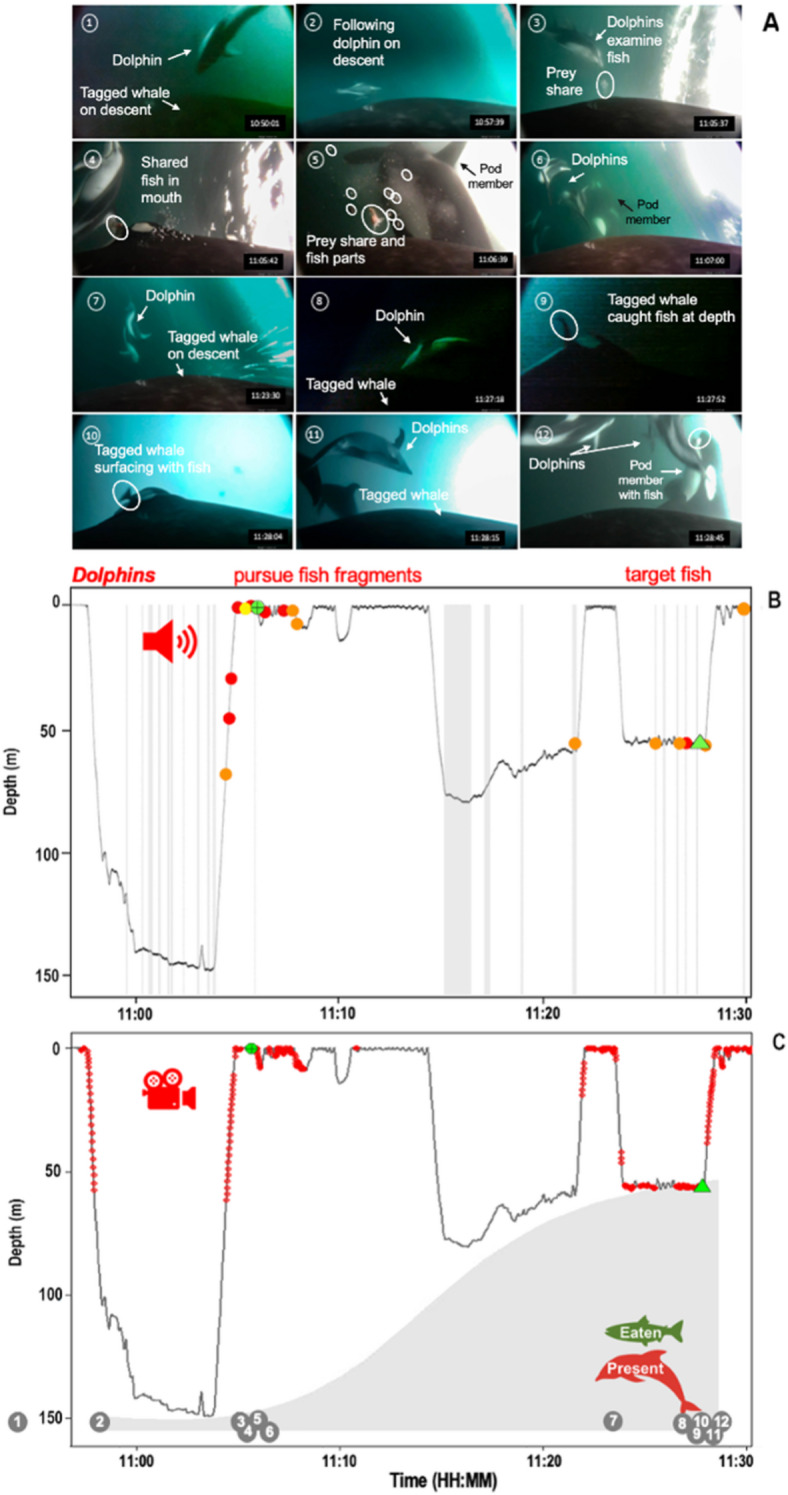
Video frame grabs, acoustic detections, and dive depths of killer whale I145 over a ~40 min period while Pacific white-sided dolphins were present. (**A**) Time-series of images showing I145 initiating the descent phase with a Pacific white-sided dolphin (Photos 1, 7), dolphins associated with another killer whale that captured a fish (6, 10), dolphins scavenging on a fish shared with I145 (3, 12), and I145 consuming the remainder of the shared fish (4). Other images show fish scales and large pieces of fish flesh lost while another killer whale consumes its kill (5), I145 successfully catching a fish while dolphins are present (9), and dolphins ascending with I145 after making a successful kill (11) and waiting for I145 at the surface (10). (**B**) Acoustic detections of dolphins (top) and killer whale I145 (bottom) by time (HH:MM PDT) separated into clicks (searching for prey), click-trains (targeted prey) and buzzes (chasing fish target). Dolphins actively produced buzzes at the surface to locate a fish being shared before scavenging it. (**C**) Corresponding dive data for I145 showing when: 1) dolphins were observed near the head of the killer whale (); 2) a fish was shared with I145 (+); 3) the tagged killer whale caught a fish (); 4). Bottom depths associated with the killer whale locations are shaded in gray. The video frame grabs were recorded on the CATs camera between 10:57:37 and 11:28:45 PDT. The successful prey capture that occurred in association with dolphins, and the subsequent consumption at the surface (11:26–11:29) is shown in Video S5.

Interspecific associations and coordinated movements were observed from aerial drone recordings (n=15 videos) totalling 84 minutes of interactions, for three tagged killer whales (I107, I145, D26) and three unidentified, non-tagged killer whales for a total of 23.3 minutes of imagery. Analysis of drone recordings (Video S1) showed killer whales followed the movements of dolphins for 25.4 cumulative minutes (at times, multiple focal whales and dolphins were recorded in the same scene resulting in a longer cumulative interaction time) and made distinct changes in orientation toward the dolphins 102 times as opposed to orienting away from the dolphins or being in front of them 18 times for 3.0 cumulative minutes. Thus, the killer whales demonstrated their preference for following the dolphins by altering their course and direction of travel to align with the dolphins’ movements, following them on foraging dives 25 times. The drone also recorded 6 occasions of dolphins actively chasing, catching, and consuming small salmon at the surface (Video S2) with killer whales nearby. Killer whales were shown with 4 successful salmon kills and held their catch for prolonged periods, totalling 3.6 minutes. During one prey capture event of a confirmed adult Chinook salmon, dolphins were observed scavenging on remains as the killer whale consumed the fish. Aerial recordings show one instance of a dolphin catching a fish, killer whales subsequently following the dolphin on a dive, which was followed by several instances of body rolling (Video S3). Although not visually confirmed, it is probable that the killer whales consumed a portion of the fish (Video S3). Although interspecies associations predominantly occurred while killer whales engaged in foraging activities (hunting and prey sharing; Figs. 3-7), only one observation (above or below water) was made of dolphins taking a free-floating piece of salmon after a killer whale ate, which may have been an intended prey share with conspecifics. The number of observations of killer whales moving away and towards dolphins was significantly different (F_1, 26_ = 17.62, p<0.001) (4) with whales turning towards dolphins more often (Fig.4). However, whether foraging attempts were successful or not had no impact on killer whale orientation (F_1, 26_ = 2.20, p=0.15).

Combining acoustic detections of crunches with visual observations of scales and prey sharing, we noted a total of 9 prey captures and 8 prey sharing events (7 where our tagged killer whale received a fish and one where our killer whale gave a fish). Of these, 7 prey captures and 4 prey shares occurred without dolphins and 2 captures, and 4 shares were in the presence of dolphins.

### Kinematics of dive behaviour

We found that kinematic variables associated with foraging^28,29^ during the bottom phase of a dive were generally comparable when dolphins were present or absent (Fig.3). However, all values marginally decreased during dolphin associations. On average mechanical work as reflected by VeDBA was 0.054 m s^−2^ ± 0.0120 SD without dolphins and 0.049 m s^−2^ ± 0.0244 SD with dolphins. The degree of change in swim heading (circular heading variance – calculated as the variance of the heading data) was 0.24 radians (± 0.112 SD) without dolphins and 0.20 radians (± 0.156 SD) with dolphins. The standard deviation of the rate of change of acceleration in during peak jerk (deviation in peak jerk) was 94 m s^−3^ (± 37.1 SD) without dolphins and 84 m s^−3^ (± 27.0 SD) with dolphins. Roll at peak jerk was 77 degrees (± 56.2 SD) without dolphins compared with 32 degrees (± 42.1 SD) with dolphins.

### Diving behaviour

Dolphins co-occurred with 4 study animals during 9% of the underwater camera recordings (Table 2). However, there was considerable variability in the proportion of total tag attachment time that the dolphins associated with individual killer whales (i.e., 21% with I145; 12% with D26; 2% with I107; and 1% with A113; Fig. 5 and Table 1). During times that dolphins were present, killer whales spent significantly less time associating with other pod members (*p*=0.004, Fisher’s exact test). Decreased spatial cohesion of killer whales belonging to the same matriline was also associated with active hunting evidenced by echolocation clicks, acceleration in speed, and abrupt direction changes. There was also a notable reduction in killer whale vocalizations (*p*=0.026, Fisher’s exact test) when dolphins were present (25.8%, n=8) compared to when they were absent (74.2%, n=23).

**Table 2:** Summary dive statistics for four northern resident killer whales (A113, I145, I107 and D26) equipped with CATs tags. Dives were separated based on videos that included dolphin associations (Dives with dolphins present) and videos where dolphins were absent (Dives with dolphins absent). Bolded values represent the average of all individual mean values (except for A113 because only 1 dive occurred when dolphins when present) for the time spent during the: 1) descent phase (Descent time), 2) bottom phase (Bottom time; time spent a depth between descent and ascent phases); 3) Ascent phase (Ascent time) of the dive, as well as the total duration of the dive (Dive dur.), the distance traveled at the bottom phase of the dive (Bottom dist.), the maximum depth of the dive (Max depth) and post-dive surface duration time (Post-dive dur). Tabulated data show means ± standard deviations, minimum and maximum values.

Animal		**Dolphins (Yes/No)**	**Descent time (secs)**	**Bottom time (secs)**	**Ascent time (secs)**	**Dive dur. (secs)**	**Bottom dist. (m)**	**Max depth (m)**	**# dives**
**Mean (± SD)**	I145	No	39.2 ± 36.96	50.76 ± 47.56	33.49 ± 19.31	123.44 ± 71.20	5.45 ± 6.26	12.91 ± 16.92	70
**Min-Max**	I145	No	7.5–311.5	1–233	3.5–90.5	16–408	0.06–26.64	3–139.40.40	
**Mean (± SD)**	I145	Yes	44.5 ± 67.84	73.25 ± 76.08	41.25 ± 47.58	159 ± 152.23	13.18 ± 16.00	39.5 ± 39.61	16
**Min-Max**	I145	Yes	3.5–282.5	1–230	3.5–184.5	8–462	0–56.82	3.06–149.39	
**Mean (± SD)**	I107	No	34.4 ± 39.29	45.1 ± 75.62	25.2 ± 29.96	104.7 ± 120.44	17.9 ± 38.97	30.4 ± 45.31	200
**Min-Max**	I107	No	2.5–260.5	1–375	05–188.5	9–511	0–243.32	3–161.34	
**Mean (± SD)**	I107	Yes	87.5 ± 110.31	33 ± 24.04	40 ± 48.79	160.5 ± 183.14	14.8 ± 16.70	55.3 ± 72.81	2
**Min-Max**	I107	Yes	9.5–165.5	16–50	5.5–74.5	31–290	3.05–26.64	3.77–106.753	
**Mean (± SD)**	D26	No	29.7 ± 21.95	31.76 ± 38.11	22.35 ± 18.63	83.81 ± 66.91	4.78 ± 11.12	11.25 ± 16.61	150
**Min-Max**	D26	No	5.5–125.5	1–174	0.5–88.5	14–285	0–96.63	3.04–147.4	
**Mean (± SD)**	D26	Yes	25.6 ± 21.95	25.36 ± 32.98	25.14 ± 25.74	76.14 ± 77.92	16.02 ± 27.89	27.5 ± 33.21	14
**Min-Max**	D26	Yes	5.5–71.5	1–113	2.5–83.5	14–255	0.06–97.56	3.17–103.28	
**Mean (± SD)**	A113	No	31.9 ± 19.71	44.6 ± 43.73	27.48 ± 21.65	104.0 ± 66.06	9.63 ± 20.75	19.21 ± 28.62	43
**Min-Max**	A113	No	5.5–77.5	2–3.237	4.5-106.5	20–361	0.16–130.2	3.1–134.85	
**Raw**	A113	Yes	21.5	16	19.5	57	4.36	19.46	1
**Average**	All	No	**33.81**	**43.05**	**27.13**	**103.98**	**9.43**	**18.43**	**463**
**Average**	All	Yes	**44.79**	**36.90**	**31.47**	**113.16**	**12.10**	**35.45**	**34**

Diving behaviour of the tagged killer whales differed based on dolphin association. Maximum dive depth (log transformed, log-likelihood ratio test, LRT=12.2, p <0.001) were deeper when dolphins were present (untransformed 34.8 m ± 37.65 SD) and shallower when absent (untransformed 20.2 m ± 33.63 SD). The distance traveled during the bottom phase (square root transformed, LRT=8.3, p =0.004) was longer when dolphins were present (untransformed 14.2 m ± 21.20 SD) and shorter when absent (untransformed 10.4 m ± 25.72 SD). No significant differences were noted in total dive duration or bottom phase duration.

### Interspecific interactions

Interspecific interactions as determined from underwater camera recordings of dolphins near the heads of the tagged killer whales (within 1–2 body lengths) occurred while the killer whales were feeding (n=14 prey consumption events, of which 7 occurred while the dolphins were present). In total, dolphins were observed near the head of the tagged killer whales 376 times when accounting for all video frames—with considerable variability between individual killer whales (94 ± 92.73 SD; 11–196 observations). The dolphins were associated with individual killer whales that actively hunted or consumed salmon—and were present during 8 successful kills at depth, and during 6 prey-sharing activities between individual killer whales near the surface. However, no interspecies interactions were observed while the tagged killer whales were engaged in non-feeding behaviours (e.g., resting, socializing, traveling).

The videos showed dolphins accompanying killer whales during the descent, bottom and ascent phases of their dives to at least 60 m, after which low light conditions prevented visual detection. However, dolphin echolocation clicks were acoustically detected at a maximum depth of 118.7 m. In total, we confirmed 87 echolocation events of dolphins (clicks, click trains and buzzes) and 147 of killer whales (clicks, click trains, buzzes, crunches and punches between the start and end times that dolphins were observed) (Table S2 & S3T). Overall, dolphin echolocation clicks were detected most frequently when tagged killer whales were foraging at 19 m (± 25.32 SD) while killer whale clicks were most often recorded at deeper depths (42.5 m ± 52.15 SD) (Fig. 6). This suggests that interspecies foraging may be more likely to occur when killer whales are hunting salmon at shallower depths.

**Figure 8:**
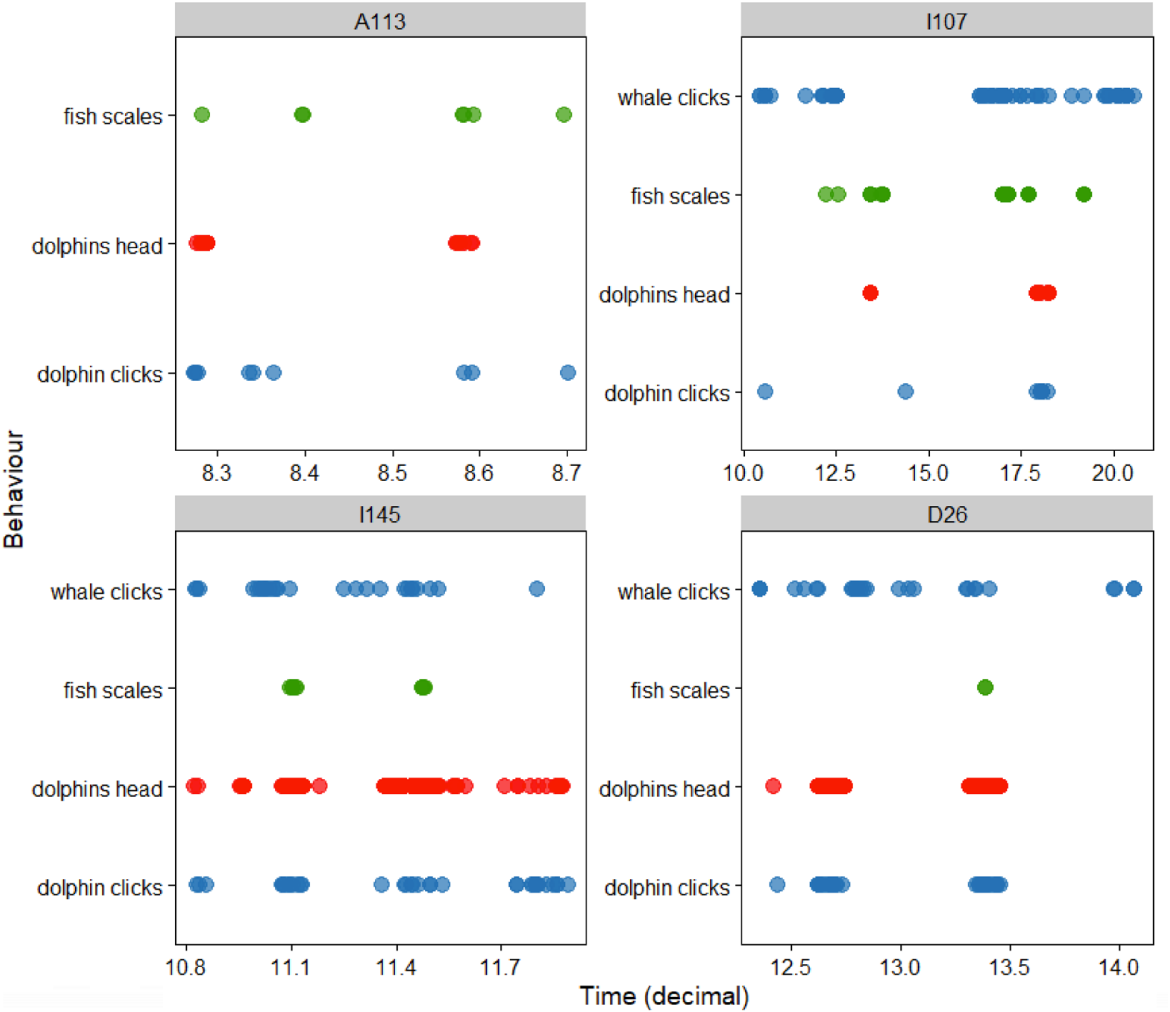
Integration of killer whale and dolphin passive acoustic and underwater camera behaviour data over time (decimal) for four northern resident killer whales (A113, I107, I145 and D26). Acoustic detections include killer whale and dolphin clicks, click trains and buzzes (blue circles) and visual observations include the presence of fish scales when a fish was consumed (green circles) and dolphins (red circles) near the head of the tagged killer whale. For the three killer whales with recorded echolocation clicks there were 18 distinct periods of activity in total and 50% of those coincided with dolphin echolocations (n=9) (Fig. 5).

Echolocations produced by three of the tagged killer whales (I107, I145 and D26) and the associated dolphins co-occurred during foraging dives but were often asynchronous in time (Figs. 5, 6, 7, 8 and Tables 3, 4). The greatest interspecific interactions involved dolphins echolocating (41.4% I145, and 34.4 % D26), and occurring in front of the killer whales (39.6%, of all I145 observations, n=149; and 52.1% of all D26 observations, n=196). Echolocation click trains comprised the greatest proportion of all acoustic detections for both killer whales (64.1%, n=84) and dolphins (52.9%, n=46) (Table 3). These click trains occurred at relatively shallow depths for D26 (12.2 m ± 22.00 SD; n=27) and its associated dolphins (11.9 m ± 20.41 SD; n=30); and at deeper depths for I145 (84.5 m ± 50.37 SD; n=26) and its associated dolphins (42.2 m ± 34.67 SD). The dolphins (n=36 observations) that associated with I145 also scanned for prey (36.7 m ± 31.66 SD clicks) and pursued targets (24.11 m ± 22.31 SD buzzes) at both shallow and deep depths following a shallow prey sharing event and a deep prey capture event (Fig. 5).

**Table 3:** Proportion of all foraging related acoustic detections made for killer whales (n=131; I107, I145 and D26) and associated dolphins (n=87) organized by type.

**Species**	**Clicks**	**Click trains**	**Buzzes**
Killer whales	32.1% (n=42)	64.1% (n=84)	3.8% (n=5)
Dolphins	3.4% (n=3)	52.9% (n=46)	43.7% (n=38)

**Table 4:** Overall duration of detected echolocation events for killer whales (I145, D26 and I107) and associated dolphins as well as the overlap duration during association. Data are separated by periods when: 1) the tagged killer whale echolocated in the absence of dolphins (killer whale dolphins absent); 2) killer whales echolocated while associated with dolphins based on visual confirmation from underwater camera footage (killer whale dolphins present); 3) dolphins echolocated near the tagged killer whale (dolphins); 4) both killer whales and dolphins echolocated at the same time (overlap between species).

Total echolocation duration (MM:SS.SSS)				
Animal	Killer whale dolphins absent	Killer whale dolphins present	Dolphins	Overlap between species
		(% of association duration)	(% association duration)	
I145	02:20.5	01:33.74 (3%)	01:55.10 (3%)	00:30.0
D26	03:28.4	02:05.15 (3%)	01:08.27 (2%)	00:06.4
I107	25:04.0	00:49.54 (19%)	00:15.15 (6%)	00:10.8

The total duration of echolocations made by D26 were reduced by 40% when dolphins were present (Table 4). D26 produced overlapping echolocations with associated dolphins 3% of the time. In contrast, I145 vocalized 33% less (total duration) when dolphins were present. Overall, all three killer whales were periodically silent when dolphins actively echolocated and showed asynchronous echolocation patterns (Figs. 5, 6, 7, 8).

## Discussion

Foraging associations between different species can reflect a wide range of ecological strategies, from direct competition to complex forms of cooperation. We therefore used a combination of aerial drone footage and biologging data to disentangle the spatial, temporal, and behavioural dynamics of associations between resident killer whales and Pacific white-sided dolphins. Our data revealed behavioural patterns that were inconsistent with competitive, kleptoparasitic or anti-predator relationships—and provided no evidence of aggression or avoidance from either species. Instead, the associations exclusively occurred during foraging events and involved coordinated movements—such as killer whales trailing dolphins—and synchronized acoustic activity. These documented behaviours suggest a functional relationship potentially rooted in cooperation.

The coordinated movements, vocalizations, prey captures and consumption events we recorded generally agree with a cooperative hunting framework^43,44^. Based on aerial drone observations, killer whales swam disproportionally towards the dolphins (n=102) and matched the dolphins’ swim path while pursuing salmon at depth (Videos 1,Videos 2,Videos 3) (n=25) and at the surface (n=6) (Video S4). Conversely, dolphins rarely pursued the swim path of killer whales at the surface (n=5 events) suggesting they infrequently follow killer whales. Consequently, dolphins may act as scouts^45^, providing foraging cues to the trailing killer whales. In this way, group foraging may enhance foraging opportunities^22,46^. However, the extent to which cooperative foraging may provide benefits for both species at relatively little cost to each is yet to be determined.

### Kleptoparasitism

The hypothesis that Pacific white-sided dolphins engage in kleptoparasitism assumes that they benefit at the killer whales’ expense by stealing prey while investing little in foraging themselves^47–49^. If true, killer whales would have been expected to display behaviours consistent with competition—such as aggression, avoidance, or other attempts to deter dolphins from accessing captured prey. However, we observed no such behaviours. Neither the aerial drone nor underwater video data revealed any signs of aggression, such as tail slaps, biting or raking appendages—as delphinids commonly do among themselves^50^.

Since kleptoparasitism can also be seen as a form of disturbance that reduces foraging efficiency, we also considered whether interspecies interactions might lead to the cessation of hunting—in the way that anthropogenic noise playback experiments can cause cetaceans to change their behaviours^51,52^. However, killer whales were also never observed fleeing or avoiding the dolphins—which are documented behavioural responses to vessel encounters^53,54^. Instead, the killer whales disproportionately oriented toward the dolphins and mirrored their swimming and diving movements (Fig. 4).

In all cases, the killer whales continued to engage in typical foraging behaviours during their interactions with the dolphins—including prey pursuit, capture, and sharing (Figs. 5, 6, 7, 8). The presence of dolphins never led to the cessation of hunting—in the way that disturbance from anthropogenic noise playback experiments can alter cetacean behaviours^51,52^. Four underwater prey-sharing events with other killer whales were documented in the presence of dolphins, and none showed evidence of theft. A single ambiguous instance of possible theft (a dolphin taking a salmon head; Video S5) was observed in the drone footage but not confirmed by simultaneous underwater recordings. Overall, the underwater footage showed no confirmed thievery despite the dolphins’ proximity to at least one shared fish (Video S1; timestamp 0:37).

Another inconsistency with the kelptoparasitism hypothesis is that the dolphins were not passive observers. A key driver of kleptoparasitism is the lack of effort required for an individual to obtain resources^47^. If kleptoparasitism was occurring, killer whales would have been expected to actively hunt salmon, while dolphins (scroungers or kleptoparasites) would have located successful killer whales without needing to be in front of the killer whales and steal their catch. Under this framework, dolphins would be contest competitors and have would obtained prey resources through thievery without allocating time and energy searching for and capturing prey^48,49^. Killer whales would in turn experience reduced resources—such as shown for California sea lions (*Zalophus californianus*) that reduce the foraging effort and success of striped marlin (*Kajikia audax*) through kleptoparasitism^55^. Instead, the biologger data and acoustic recordings revealed the dolphins actively hunting during associations (for a cumulative average of 8.2 mins ± 7.65 SD), making deep dives (exceeding 60 m) and producing echolocation buzzes consistent with targeting prey (Videos 4 and 5; Figs. 5 and 7). These patterns suggest the dolphins and killer whales were both investing effort in locating and capturing prey. The absence of theft or aggression, coupled with parallel foraging efforts, indicates that kleptoparasitism is not the primary mechanism underlying these associations.

### Reduced predation risk

Another potential, although speculative, reason why Pacific white-sided dolphins and resident killer whales associate is to obtain protection from mammal-eating transient killer whales^6^. Mammal-eating (transient or Bigg’s) killer whales are predators of Pacific white-sided dolphins and occupy overlapping coastal regions with fish-eating (northern resident) killer whales along the coast of British Columbia, Canada^13^. However, these two distinct ecotypes generally avoid each other, both spatially and acoustically^13,56^—suggesting that the presence of resident killer whales could serve as a refuge from transient predation pressure while the dolphins forage on fish species such as Pacific herring (*Clupea harengus*) that are not targeted by resident killer whales.

Pacific white-sided dolphins could theoretically exploit the ecological separation between killer whale ecotypes^8,57,58^ to obtain incidental protection by feeding on herring in areas where resident killer whales occur and transient killer whales are less likely to be present. Dolphins can presumably distinguish between ecotypes based on the well-documented differences in acoustic cues of resident and transient killer whales^14^, thereby enabling them to make informed choices about which killer whales to associate with. Non-predatory, aerial observations (n=42) of northern resident killer whales interacting with Dall’s porpoise and Pacific white-sided dolphins while engaged in non-foraging behaviours (e.g., travelling, milling, socializing and resting) suggest adaptive antipredatory benefits of interspecies association, and possibly hydrodynamic benefits as well^7^. Though speculative, protective association could explain at least part of the behavioural tolerance observed between dolphins and resident killer whales, even in the absence of direct foraging benefits.

Another intriguing hypothesis is that the dolphins spend time engaging with resident killer whales to learn anti-predator behaviours. Unfortunately, our acoustic and behavioural analyses were not designed to evaluate the development of anti-predator behaviours. Instead, our data point more clearly to dolphins engaging in active foraging for adult Chinook during their direct associations with northern resident killer whales (evidenced by confirmed salmonid captures at the surface by drone and production of fast clicks and buzzes while associated with foraging killer whales at depth), indicating that any predation risk reduction is likely a secondary, indirect benefit rather than a primary driver of the relationship. Nevertheless, the potential for a predatory refuge effect warrants further investigation, particularly through studies incorporating transient killer whale presence and dolphin response behaviours.

### Cooperative foraging

The coordinated foraging behaviours exhibited by resident killer whales and Pacific white-sided dolphins suggests they were not merely tolerating each other but engaged in an opportunistic cooperative partnership. All the associations we documented between the two species occurred exclusively in the context of foraging, and all were undertaken with a high degree of behavioural alignment.

Cooperative foraging is characterized by individuals working together to detect, pursue, and capture prey, often resulting in enhanced efficiency or success compared to solitary efforts^46,59^. This behaviour can occur along a spectrum of complexity, from loosely aligned hunting efforts to highly coordinated or even collaborative roles^60^. In its simplest form, animals exhibit behavioural similarity by targeting the same prey in close proximity without spatial or temporal coordination. In contrast, synchronous hunting involves individuals using similar strategies simultaneously, while coordinated hunting includes both spatial and temporal alignment^60^. The most complex form of cooperative foraging—collaborative hunting—involves individuals performing distinct but complementary roles to capture the same prey^60^.

While various forms of cooperative foraging are well documented within species—particularly among delphinids employing diverse tactics tailored to prey type, habitat, and social structure^16–18^—interspecific cooperation is far less common^20^, especially among marine mammals^1,2^. When mixed species foraging groups are identified, it is not uncommon for one species to form a leadership role (i.e., nuclear species) with another species following behind^46^. Such following behaviour is a hallmark of interspecific foraging for reef sharks^61^. In this instance, species that engage in following behaviour benefit from new feeding opportunities through the leader flushing prey (invertebrates and fishes)^61^. Biologgers with cameras have also recorded nurse sharks following benthic feeding bottlenose dolphins and filter feeding near them, likely to consume prey stirred up by the dolphins and improve their foraging opportunities^79^. We similarly found evidence of dolphin leadership as tagged killer whales trailed behind dolphins during all phases of their foraging dives.

Our data suggest that the documented associations between resident killer whales and Pacific white-sided dolphins were more than incidental co-occurrences. The aerial drone observations revealed the killer whales disproportionately orienting toward and swimming in alignment with dolphins (n = 102), mirroring their paths during pursuits of salmon at both depth (n = 25) and near the surface (n = 6). Conversely, the dolphins only followed the swim paths of killer whales on five occasions, suggesting that the dolphins were not simply trailing in hopes of feeding opportunities. Instead, this directional bias supports the hypothesis that the dolphins served as mobile information sources—akin to “scouts”—that facilitated prey detection^45^. These behaviours indicate a level of coordination consistent with the coordination or potentially collaboration levels of cooperative hunting^60^, in which individual actions are spatiotemporally related and may contribute uniquely to shared foraging success. In this way, group hunting may enhance foraging opportunities^46,79^. However, the extent to which cooperative foraging provides benefits for both species at relatively little cost to each is yet to be determined.

A potential mechanism supporting the apparent coordination is acoustic cue-sharing, given that both species have overlapping hearing ranges (killer whales: ~1–100 kHz; dolphins: ~0.75–150 kHz)^62,63^ that allows for reciprocal perception of echolocation clicks and buzzes. We documented simultaneous and alternating echolocations by both species (Figs. 6, 7, 8, S1), suggesting they engaged in concurrent prey searching. The killer whales may have also reduced their own echolocation effort to benefit from the biosonar activity of dolphins—a form of interspecific eavesdropping similar to behaviour seen in Cuvier’s beaked whales and echolocating bats^64,65^. Multiple scanning dolphins may thus act as a distributed sensor array, increasing detection efficiency for prey such as the large and fast-swimming adult Chinook salmon.

In addition to echolocation, spatial foraging patterns further support the hypothesis of cooperative foraging. Most notably, the killer whales dove to greater depths when associated with the dolphins (mean depth = 35 m) compared to when alone (mean = 20 m), and they traveled longer distances, possibly reflecting an expanded search area enabled by dolphin cues. The killer whales also demonstrated reduced body rolls during prey pursuits when the dolphins were present (77-degree without and 32-degree roll with dolphins), potentially reflecting changes in acoustic search strategies. Since odontocetes have a narrow acoustic field of view^66^ (i.e., conically shaped biosonar beam), increased body roll (e.g., 21-degrees on average while foraging) has been proposed to improve the detection range of foraging resident killer whales^29^. Such behavioural shifts suggest that the killer whales we tracked relied on dolphin echolocation activity to refine their search for migrating adult Chinook salmon, which typically migrate closer to the surface, but can evade predators by hiding at depth^67,68^.

Resident killer whales primarily target large, adult Chinook salmon ranging from ~80 cm and 8.5 kg at 4 years of age to over 95 cm and 13.5 kg at 6 y years^8,69,70^. In contrast, Pacific white-sided dolphins consume predominately smaller (15 cm) prey such as herring (59%), followed by pink, sockeye, and coho salmon (*Oncorhynchus spp;* 30%) up to 60 cm as determined from aged scales recovered at the surface^11^. In contrast, the largest fish recovered from dolphin stomachs have been Pacific hake (*Merlucclus productus*) measuring 40–50 cm^10^. There is no evidence that Pacific white-sided dolphins can break up large fish such as adult Chinook salmon, into smaller pieces prior to ingestion^10^.

Killer whales use a grip-and-tear feeding method to process adult Chinook salmon, often biting fish apart to share with pod members, which releases tissue, scales and blood into the surrounding water^8,71,72^. Pacific white-sided dolphins, on the other hand, are raptorial feeders that swallow prey whole and are not anatomically equipped to dismember large fish. This fundamental difference in feeding mechanics may allow the killer whales and dolphins to share parts of large prey without competing with each other (i.e., resource partitioning)—thereby enabling the dolphins to exploit scraps left by the killer whales (Videos 5 and S1). This difference in feeding capabilities may also explain the rare occasions we observed dolphins briefly holding and subsequently releasing large salmon near the surface (Video S2), which could incidentally aid nearby killer whales in locating, capturing and breaking apart prey.

Hydroacoustic surveys of the areas where the dolphins and killer whales co-occurred revealed relatively low densities of adult Chinook and high abundances of Pacific herring^67^. It is likely that both the Chinook and dolphins were feeding on the herring. In British Columbia, Chinook salmon have a varied diet that includes small schooling fishes such as Pacific herring, northern anchovy and sand lance, as well as invertebrates like euphausiids^73,74^. These prey preferences create conditions where dolphins, Chinook, and resident killer whales overlap in space—potential increasing opportunities for interactions between the dolphins and killer whales.

Hydroacoustic survey data for adult Chinook, along with independent acoustic fish tracking data, suggest that adult Chinook typically migrate near the surface (mean depth ~22 m), which aligns with the primary dive depths of foraging killer whales (10–30 m)^67,68,75^. It appears that migrating Chinook generally avoid deeper depths unless actively evading predators^27,76^, in which case the dolphins may assist killer whales in scanning areas at depth resulting in expanded spatial search coverage. Such coordinated behaviour is presumably reinforced by both species receiving food rewards and may be more prevalent during summer and fall when adult Chinook are migrating. However, the extent to which dolphins obtain reciprocal energetic benefits by accessing fish scraps is yet to be determined. It is also unknown whether this cooperative relationship occurs at other times of the year when Chinook are less available.

Whether similar cooperative foraging interactions occur between another salmon-specialist—the southern resident killer whales—and Pacific white-sided dolphins remains an open question. However, it is plausible that the cooperative foraging strategy we observed is a culturally transmitted behaviour that is exclusive to northern residents—similar to the kelp allogrooming behaviour documented among southern residents^77^ that contrasts with the beach-rubbing traditions of northern resident killer whales^58^. Southern resident killer whales have also been reported to regularly harass and kill harbour (*Phocoena phocoena*) and Dall’s porpoise (*Phocoenoides dalli*), which has been rarely observed among northern and Alaskan resident killer whale populations^78^, further underscoring the potential for population-specific cultural behaviours. Continued observation and long-term study are required to uncover the extent and significance of such behavioural differences between killer whale populations.

## Conclusions

Given the size mismatch between adult Chinook and the feeding capabilities of dolphins, it is likely that the dolphins were primarily in our study area to exploit herring and opportunistically associated with the foraging killer whales when there was an opportunity for them to participate in hunts that provided access to salmonid prey remains. While the energetic payoff of such associations may be small, the lack of competitive or antagonistic behaviour suggests mutual tolerance that yields reciprocal benefit for both species. However, determining the extent to which coordinated hunting provides mutual benefits to dolphins and killer whales requires further tagging studies to quantify the impact of cooperation on foraging effort and success.

Our analysis of killer whale movement, foraging success and interactions with allospecifics provides new understanding of interspecies interactions among marine mammals. Coordinated predation between northern resident killer whales and Pacific white-sided dolphins may reduce echolocation time and enhance prey detection and capture success. It is also possible that the presence of resident killer whales affords some degree of refuge from predation by transient killer whales. While the full extent and reciprocity of benefits remain to be quantified, our data suggest that interspecific cooperative foraging offers ecological advantages to both species, particularly during seasonal Chinook salmon migrations when prey is more concentrated but also more evasive. Whether this behavioural pattern persists during other seasons or in the absence of high-value prey remains an open question and warrants further investigation.

## Supplementary Information

Supplementary Information 1.

Supplementary Video 1.

Supplementary Video 2.

Supplementary Video 3.

Supplementary Video 4.

Supplementary Video 5.

## Data Availability

The datasets used and/or analysed during the current study available from the corresponding author on reasonable request.
