# Supplementary material for: Cooperative foraging between dolphins and fish-eating killer whales
Source: Sci Rep. 2025 Dec 11;15:42897. doi: 10.1038/s41598-025-22718-4 (PMC12698851; doi:10.1038/s41598-025-22718-4)
Supplement: Supplementary file 1 — Supplementary Information 1. [file 41598_2025_22718_MOESM1_ESM.pdf]

Supplementary Materials for

Cooperative foraging between fish-eating killer whales and dolphins

Sarah M. E. Fortune, Xi Cheng, Keith Holmes and Andrew W. Trites

Correspondence to: sarah.fortune@dal.ca

Figs. S1
Tables S1 to S3
Videos S1 to S5

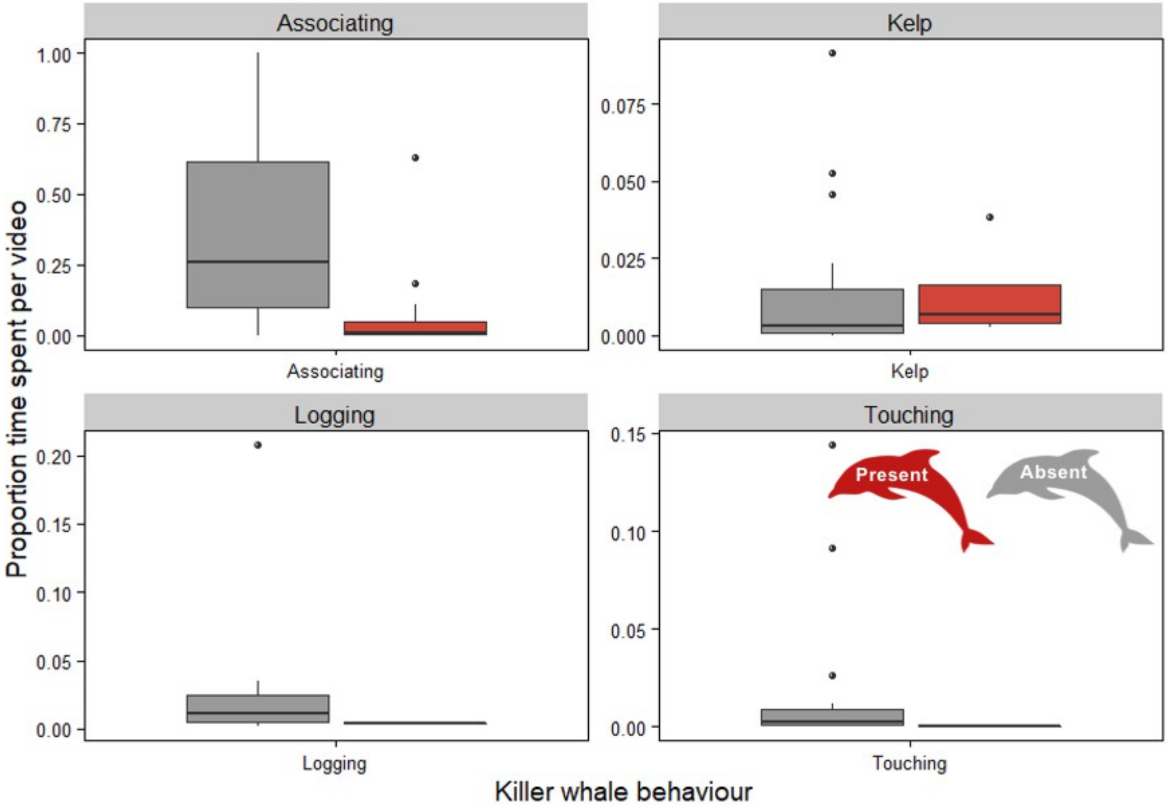

Figure S1. The proportion of time tagged northern resident killer whales engaged in five

common behaviors while dolphins were present or absent based on ethograms derived from underwater video analysis. Behavioral states included: 1) the tagged whale associating with other killer whales (Associating); 2) swimming through and physically interacting with kelp (Kelp); 3) resting at the surface (Logging); and 4) touching other whales (Touching).

Table S1. Behavioural observations made from CATs underwater video and drone video recordings using BORIS. Whether the observation occurred at a discrete time (Point) or over a period of time (State) is indicated by 'Type'. A 'description' of the observation is provided along with a general 'behavioural category' that discerns acoustically detected behavioural observations associated with social and foraging activity, from visually observed active, foraging, interspecies, movement, rubbing, social and other behaviours.

Observation	Type	Description	Behavioural category	CATS / Drone
Vocalization response	Point	tagged whale vocalizes after another killer whale calls	Acoustics (social)	C
Vocalization	State	vocalizations are detected	Acoustics (social)	C
Echolocation	Point	echolocation clicks are detected	Acoustics (foraging)	C
Crunch	Point	biting a fish with audible 'crunch' detected	Acoustics (foraging)	C
Scales	Point	scales, flesh and/or clouds of blood	Foraging	C
Dolphins head	State	dolphin(s) near the head of tagged killer whale	Interspecies	C
Surface	State	ascent is completed as whale breaks the surface	Movement	C
Quick	Point	an increase in flow noise is acoustically detected	Movement	C
Tag orientation	Point	the position of the tag has changed	Other	C
Associated with whale	Point	tagged whale is traveling with another killer whale	Other	C
Bottom	State	the seafloor is visible on video	Other	C
Visibility	State	light level is low and the video is black	Other	C
Rubbing	Point	killer whale rub's body along sea bottom	Rubbing	C
Touching	Point	killer whale rub's body against another whale	Rubbing	C
Spy hop	Point	in vertical position and head is above water	Active	C / D
Breach	Point	killer whale jumps out of water	Active	C / D
Tail slap	Point	slaps tail on surface of water	Active	C / D
Pectoral slap	Point	slaps pectoral fin on surface of water	Active	C / D
Kill	Point	caught and killed a fish	Foraging	C / D

Confirmed Fish Capture	Point	full fish in mouth but kill not observed	Foraging	C / D
Share_Giver	Point	shared fish with another killer whale	Foraging	C / D
Share_Recipient	Point	fish was shared with tagged killer whale	Foraging	C / D
Dolphins	Point	dolphin(s) present with tagged killer whale	Interspecies	C / D
Dolphins scavenge	Point	dolphin(s) consume pieces of fish	Interspecies	C / D
Porpoises	Point	Dall's porpoise(s) present	Interspecies	C / D
Logging	Point	nearly motionless and is resting at the surface	Movement	C / D
Dive	State	descent phase of dive has begun	Movement	C / D
Orientation killer whale	Point	Killer whale changes orientation (on side/upside-down)	Movement	C / D
Kelp	Point	playing in kelp bed	Other	C / D
Killer whale with salmon	State	killer whale with salmon in mouth	Foraging	D
Killer whale orients towards dolphin	State	killer whale orienting towards dolphin - Dolphin(s) takes the lead (with or without intention)	Movement	D
Killer whale orients away from dolphin	State	killer whale orienting away from dolphin(s) - avoid	Movement	D
Killer whale takes lead	State	killer whale takes the lead and dolphin(s) follow	Movement	D
Dolphin chase salmon	Point	dolphin chasing salmon near surface with killer whales nearby	Foraging	D
Chase	State	killer whale is chasing a fish	Foraging	D
Killer whale aggression	Point	killer whale aggression (e.g., charging, biting) towards dolphins	Active	D

Table S2. Summary of all foraging related passive acoustic detections made for four northern resident killer whales (A113, I145, I107 and D26) equipped with CATs tags. The type of acoustic detection is defined whereby clicks and click trains reflect animals that are scanning the environment for prey. Fast clicks and buzzes occur when animals have honed into a fish target.The detection of punch sounds indicates the whale was in close contact with a fish (either caught or near capture) and crunch sounds indicate the whale is actively consuming a fish. The 'start' and 'stop' times (HH:MM:SS) are in PDT and 'duration' reflects the time spent engaged in each type of acoustic behavior. Using underwater video and passive acoustic detections of dolphins when light levels are too low to permit visual confirmation, we indicate whether 'dolphins' were present (yes) or absent (no).

Animal	Type	Start	Stop	Duration	Dolphins
I145	click train (slow down)	10:49:29	10:49:40	00:00:12	Yes
I145	click train	10:49:49	10:49:54	00:00:06	Yes
I145	click train	10:50:12	10:50:20	00:00:08	Yes
I145	punch	10:59:29	NA	NA	Yes
I145	click train	10:59:30	10:59:32	00:00:02	Yes
I145	click train	11:00:16	11:00:19	00:00:02	Yes
I145	click train	11:00:37	11:00:48	00:00:10	Yes
I145	click train	11:01:05	11:01:10	00:00:05	Yes
I145	click train	11:01:34	11:01:37	00:00:03	Yes
I145	punch	11:01:41	NA	NA	Yes
I145	click train	11:01:42	11:01:47	00:00:05	Yes
I145	click train	11:02:19	11:02:20	00:00:01	Yes
I145	punch	11:02:59	NA	NA	Yes
I145	click train	11:03:00	11:03:05	00:00:05	Yes
I145	click train	11:03:31	11:03:34	00:00:03	Yes
I145	click train	11:03:46	11:03:52	00:00:06	Yes
I145	click train	11:05:49	11:05:50	00:00:01	Yes
I145	click train	11:15:08	11:16:24	00:01:16	Yes
I145	click train	11:17:06	11:17:21	00:00:15	Yes
I145	click train	11:18:53	11:18:59	00:00:06	Yes
I145	click train	11:21:25	11:21:36	00:00:11	Yes
I145	click train	11:25:28	11:25:31	00:00:03	Yes
I145	click train	11:25:52	11:25:57	00:00:05	Yes
I145	click train	11:26:32	11:26:35	00:00:03	Yes
I145	click train	11:26:57	11:26:59	00:00:02	Yes
I145	click train	11:27:31	11:27:33	00:00:01	Yes
I145	punch	11:27:49	NA	NA	Yes
I145	click train	11:29:47	11:29:52	00:00:04	Yes
I145	click train	11:31:17	11:31:52	00:00:35	Yes
I145	click train	11:48:11	11:48:45	00:00:34	Yes
D26	click train	12:21:24	12:21:24	00:00:00	No
D26	click train	12:21:27	12:21:27	00:00:00	No

D26	click train	12:21:29	12:21:29	00:00:00	No
D26	click train	12:30:49	12:30:50	00:00:00	Yes
D26	click train	12:33:26	12:33:26	00:00:00	Yes
D26	click train	12:36:55	12:37:03	00:00:09	Yes
D26	click train	12:37:11	12:37:31	00:00:20	Yes
D26	click train	12:46:22	12:46:34	00:00:12	Yes
D26	click train	12:46:45	12:47:01	00:00:16	Yes
D26	click train	12:47:28	12:47:39	00:00:12	Yes
D26	click train	12:47:45	12:48:08	00:00:23	Yes
D26	click train	12:48:13	12:48:30	00:00:17	Yes
D26	click train	12:49:06	12:49:34	00:00:29	Yes
D26	click train	12:49:49	12:49:58	00:00:09	Yes
D26	click train	12:50:24	12:50:47	00:00:22	Yes
D26	click train	12:59:30	12:59:43	00:00:13	Yes
D26	click train	13:02:00	13:02:07	00:00:07	Yes
D26	click train	13:03:23	13:03:31	00:00:08	Yes
D26	click train	13:18:05	13:18:14	00:00:09	Yes
D26	click train	13:18:28	13:18:54	00:00:26	Yes
D26	click train	13:20:14	13:20:29	00:00:15	Yes
D26	click train	13:20:32	13:20:53	00:00:21	Yes
D26	crunch	13:23:01	NA	NA	Yes
D26	crunch	13:23:50	NA	NA	Yes
D26	click train	13:24:26	13:24:28	00:00:02	Yes
D26	click train	13:58:11	13:58:22	00:00:12	No
D26	click train	13:58:33	13:58:47	00:00:14	No
D26	click train	14:03:52	14:03:53	00:00:01	No
D26	click train	14:03:54	14:03:59	00:00:05	No
I107	clicks	10:26:41	10:26:44	00:00:04	No
I107	click train	10:26:50	10:27:02	00:00:13	No
I107	click train	10:35:10	10:35:11	00:00:01	No
I107	click train	10:35:11	10:35:13	00:00:01	No
I107	click train	10:35:13	10:35:14	00:00:01	No
I107	click train	10:35:15	10:35:16	00:00:02	No
I107	click train	10:35:25	10:35:25	00:00:01	No
I107	click train	10:42:52	10:42:53	00:00:01	No
I107	click train	11:40:53	11:40:58	00:00:05	No
I107	click train	12:07:29	12:07:50	00:00:21	No
I107	click train	12:08:15	12:08:18	00:00:03	No
I107	buzz	12:08:46	12:08:46	00:00:01	No
I107	crunch	12:14:16	NA	NA	No
I107	click train	12:22:37	12:23:28	00:00:52	No
I107	click train	12:23:39	12:24:01	00:00:22	No
I107	click train	12:24:03	12:24:22	00:00:19	No
I107	click train	12:26:37	12:27:40	00:01:03	No
I107	click train	12:27:42	12:28:13	00:00:31	No

I107	click train	12:28:14	12:28:58	00:00:44	No
I107	click train	12:30:58	12:31:07	00:00:09	No
I107	click train	12:31:51	12:31:54	00:00:03	No
I107	click train	12:32:02	12:32:12	00:00:10	No
I107	crunch	12:33:22	NA	NA	No
I107	crunch	13:44:12	NA	NA	No
I107	clicks	16:23:52	16:24:04	00:00:12	No
I107	clicks	16:24:09	16:24:27	00:00:18	No
I107	clicks	16:28:42	16:29:02	00:00:21	No
I107	clicks	16:29:07	16:29:26	00:00:18	No
I107	clicks	16:30:50	16:31:02	00:00:12	No
I107	clicks	16:36:50	16:36:54	00:00:04	No
I107	clicks	16:43:53	16:43:59	00:00:06	No
I107	clicks	16:44:04	16:44:06	00:00:02	No
I107	clicks	16:44:11	16:44:18	00:00:07	No
I107	clicks	16:44:23	16:44:29	00:00:06	No
I107	clicks	16:44:34	16:44:38	00:00:04	No
I107	click train	16:54:15	16:54:21	00:00:06	No
I107	click train	16:55:26	16:55:30	00:00:05	No
I107	click train to buzz	16:54:49	16:54:59	00:00:09	No
I107	click train to buzz	16:55:33	16:55:42	00:00:10	No
I107	click train	16:56:25	16:57:06	00:00:41	No
I107	click train	16:57:35	16:58:36	00:01:01	No
I107	crunch	16:59:06	NA	NA	No
I107	click train	17:04:26	17:05:07	00:00:41	No
I107	click train	17:05:13	17:05:21	00:00:08	No
I107	click train	17:05:23	17:05:42	00:00:19	No
I107	click train	17:05:47	17:05:57	00:00:09	No
I107	click train	17:06:03	17:06:06	00:00:03	No
I107	buzz to click train	17:06:09	17:06:39	00:00:30	No
I107	crunch	17:07:48	NA	NA	No
I107	punch	17:09:18	NA	NA	No
I107	crunch	17:09:32	NA	NA	No
I107	click train	17:15:35	17:15:37	00:00:02	No
I107	clicks	17:29:22	17:29:42	00:00:20	No
I107	clicks	17:29:47	17:29:59	00:00:13	No
I107	click train	17:39:58	17:40:04	00:00:06	No
I107	crunch	17:40:50	NA	NA	No
I107	crunch	17:42:15	NA	NA	No
I107	crunch	17:42:35	NA	NA	No
I107	clicks	17:55:53	17:56:07	00:00:14	Yes
I107	clicks	17:56:34	17:56:38	00:00:04	Yes
I107	clicks	18:03:06	18:03:32	00:00:26	Yes
I107	clicks	18:14:32	18:14:37	00:00:05	Yes

I107	buzz	18:52:20	18:52:21	00:00:00	No
I107	click train	19:11:47	19:11:53	00:00:07	No
I107	clicks	19:46:25	19:46:35	00:00:10	No
I107	clicks	19:46:42	19:47:13	00:00:31	No
I107	clicks	19:52:50	19:53:12	00:00:22	No
I107	clicks	19:53:16	19:53:34	00:00:18	No
I107	clicks	20:05:10	20:06:34	00:01:25	No
I107	clicks	20:06:45	20:06:51	00:00:06	No
I107	clicks	20:06:57	20:07:16	00:00:20	No
I107	clicks	20:07:21	20:07:44	00:00:23	No
I107	clicks	20:07:52	20:08:09	00:00:17	No
I107	clicks	20:08:15	20:08:38	00:00:23	No
I107	clicks	20:08:43	20:09:14	00:00:31	No
I107	clicks	20:09:21	20:09:37	00:00:17	No
I107	clicks	20:09:43	20:10:03	00:00:20	No
I107	clicks	20:11:49	20:11:58	00:00:09	No
I107	clicks	20:20:41	20:20:47	00:00:06	No
I107	clicks	20:20:54	20:21:01	00:00:07	No
I107	clicks	20:21:06	20:21:17	00:00:10	No
I107	clicks	20:21:21	20:21:28	00:00:07	No
I107	clicks	20:21:33	20:21:39	00:00:06	No
I107	clicks	20:21:45	20:21:55	00:00:10	No
I107	clicks	20:22:01	20:22:07	00:00:06	No
I107	clicks	20:22:13	20:22:18	00:00:05	No
I107	clicks	20:22:44	20:33:39	00:10:55	No
I107	clicks	20:33:42	20:33:50	00:00:08	No

Table S3. Summary of all foraging related passive acoustic detections made for dolphins that were associated with northern resident killer whales (A113, I145, I107 and D26) equipped with CATs tags. 'Associated whale' indicates which animal the dolphin(s) were associated with during the time of acoustic recording. The 'type' of acoustic detection is the same for Table S4 but excludes punch and crunch sounds as these were not detected for dolphins. The 'start' and 'stop' times (HH:MM:SS) are in PDT and 'duration' reflects the time spent engaged in each type of acoustic behavior.

Associated whale	Type	Start	Stop	Duration
I145	click train	10:49:45	10:49:56	00:00:10
I145	click train	10:50:19	10:50:21	00:00:02
I145	click train	10:51:32	10:51:35	00:00:03
I145	click train	11:04:22	11:04:26	00:00:04
I145	fast click train to buzz	11:04:31	11:04:36	00:00:04
I145	fast click train to buzz	11:04:37	11:04:38	00:00:01
I145	buzz	11:04:58	11:04:59	00:00:01
I145	fast click train to buzz	11:05:35	11:05:39	00:00:04
I145	clicks	11:05:41	11:05:43	00:00:02
I145	fast click train to buzz	11:06:16	11:06:18	00:00:01
I145	buzz to fast click train	11:07:12	11:07:15	00:00:03
I145	click train	11:07:40	11:07:43	00:00:03
I145	click train	11:07:54	11:07:55	00:00:01
I145	click train	11:21:30	11:21:34	00:00:04
I145	click train	11:25:28	11:25:30	00:00:02
I145	clicks	11:25:40	11:25:40	00:00:01
I145	click train	11:26:37	11:26:41	00:00:04
I145	fast click train to buzz	11:26:57	11:26:58	00:00:00
I145	click train	11:27:53	11:28:05	00:00:13
I145	fast click train to buzz	11:29:47	11:29:48	00:00:01
I145	click train	11:29:50	11:29:52	00:00:02
I145	clicks	11:31:50	11:31:51	00:00:01
I145	fast click train to buzz	11:44:36	11:44:39	00:00:03
I145	fast click train to buzz	11:44:43	11:44:45	00:00:02
I145	fast click train to buzz	11:47:10	11:47:16	00:00:06
I145	buzz	11:47:21	11:47:26	00:00:05
I145	buzz	11:47:53	11:47:55	00:00:03
I145	fast click train to buzz	11:47:56	11:47:58	00:00:02
I145	fast click train to buzz	11:48:07	11:48:08	00:00:01
I145	fast click train to buzz	11:48:12	11:48:18	00:00:06
I145	fast click train to buzz	11:48:22	11:48:30	00:00:07
I145	fast click train to buzz	11:49:45	11:49:50	00:00:06
I145	fast click train to buzz	11:50:57	11:51:02	00:00:06
I145	click train	11:51:32	11:51:33	00:00:01
I145	buzz	11:51:35	11:51:37	00:00:02
I145	click train	11:53:24	11:53:25	00:00:01

D26	click train	12:26:17	12:26:19	00:00:01
D26	fast click train to buzz	12:37:11	12:37:14	00:00:03
D26	click train	12:37:20	12:37:21	00:00:01
D26	click train	12:37:29	12:37:31	00:00:02
D26	click train	12:37:54	12:37:56	00:00:02
D26	fast click train to buzz	12:38:22	12:38:27	00:00:06
D26	click train	12:39:30	12:39:31	00:00:01
D26	click train	12:40:25	12:40:26	00:00:01
D26	fast click train to buzz	12:40:39	12:40:42	00:00:03
D26	buzz	12:40:43	12:40:44	00:00:01
D26	click train	12:41:02	12:41:03	00:00:02
D26	click train	12:41:04	12:41:04	00:00:01
D26	fast click train to buzz	12:41:24	12:41:26	00:00:03
D26	click train	12:42:06	12:42:13	00:00:08
D26	click train	12:42:19	12:42:20	00:00:01
D26	buzz	12:44:04	12:44:06	00:00:02
D26	click train	13:20:31	13:20:33	00:00:02
D26	click train	13:21:11	13:21:13	00:00:02
D26	fast click train to buzz	13:21:35	13:21:36	00:00:01
D26	click train	13:22:10	13:22:10	00:00:00
D26	click train	13:22:18	13:22:28	00:00:10
D26	click train	13:23:03	13:23:04	00:00:02
D26	click train	13:23:05	13:23:08	00:00:03
D26	click train	12:23:43	12:23:45	00:00:02
D26	click train	12:24:09	12:24:15	00:00:06
D26	click train	13:25:25	13:25:27	00:00:03
D26	click train	13:26:01	13:26:02	00:00:01
D26	click train	13:26:15	13:26:21	00:00:06
D26	click train	13:26:25	13:26:30	00:00:04
D26	click train	13:27:06	13:27:07	00:00:01
A113	click train	08:16:28	08:16:29	00:00:01
A113	click train	08:16:31	08:16:32	00:00:01
A113	click train	08:16:36	08:16:38	00:00:01
A113	click train	08:16:43	08:16:45	00:00:02
A113	click train	08:20:18	08:20:20	00:00:02
A113	click train	08:20:33	08:20:35	00:00:02
A113	buzz	08:21:56	08:21:56	00:00:01
A113	click train to buzz	08:34:58	08:34:59	00:00:01
A113	click train	08:35:29	08:35:30	00:00:01
A113	click train	08:42:01	08:42:02	00:00:01
I107	buzz	10:34:14	10:34:15	00:00:00
I107	click train	14:23:41	14:23:44	00:00:02
I107	fast click train to buzz	17:56:08	17:56:09	00:00:01
I107	buzz	18:03:18	18:03:19	00:00:01

I107	fast click train to buzz	18:03:19	18:03:23	00:00:04
I107	buzz	18:03:24	18:03:25	00:00:01
I107	buzz	18:03:25	18:03:28	00:00:03
I107	fast click train to buzz	18:03:28	18:03:29	00:00:01
I107	fast click train to buzz	18:03:29	18:03:31	00:00:01
I107	fast click train to buzz	18:03:31	18:03:33	00:00:02
I107	click train	18:14:10	18:14:12	00:00:01

https://drive.google.com/file/d/15Ir8EETHTb_IjJGbaP84aADTLg6n085G/view?t=36

Video S1. Aerial video of a tagged killer whale orienting towards and following behind two Pacific white-sided dolphins at the surface and on a dive (August 30, 2020).

https://drive.google.com/file/d/1GvawG9Z3Ie_hNOKSf6DLtc82Fyz4UQos/view?usp=drive_link

Video S2. Aerial video of coordinated horizontal movement between Pacific white-sided dolphins and killer whales. Two dolphins are observed chasing salmon at the surface (August 30, 2020) ahead of two northern resident killer whales. A dolphin successfully catches a salmon (0:011) following a splash at the surface (0:09). Dolphin loses fish (0:20) and quickly turns direction and resume chasing. A killer whale is seen following behind another dolphin near the surface (0:30) and follows the dolphin on a dive (0:35). Killer whale surfaces after dolphin (0:55) and resumes diving (1:00).

https://drive.google.com/file/d/1plUzWQ4QgLWDPWTls5CvxshcRWKW7O7s/view

Video S3. Aerial video of two killer whales diving to intercept two Pacific white-sided dolphins after the capture of a salmon (0:20). Two dolphins are observed chasing salmon at the surface (August 30, 2020) ahead of two northern resident killer whales. The killer whales orient themselves in the direction of the hunting dolphins and follow the dolphins to depth.

https://drive.google.com/file/d/1fm31laYZZJtjqKHHzFb0k0J4_3dDl60w/view?usp=drive_link

Video S4. Underwater video from D26 between 13:22:18 and 13:23:09 PDT (Aug 31, 2020) during which the tagged killer whale unsuccessfully searches for a fish with accompanying dolphins, making brief but audible echolocation clicks (13:22:18) and receiving a portion of a fish shared by another killer whale (13:22:59). Distinct acoustic crunches are heard as fish fragments escape the killer whale's mouth (13:23:01), and dolphins intermittently swim near the head of the killer whale. Corresponding depth and activity profiles recorded during filming are shown in Fig. 1.

https://drive.google.com/file/d/1qosroDeUPrdoQY5-V3bbKcfkKM4Ycs3b/view?usp=drive_link

Video S5. Underwater video from I145 between 11:23:37 and 11:24:50 PDT (Aug 30, 2020) during which the tagged whale follows a dolphin to depth during a foraging dive (11:23:37). The whale vocalizes (11:23:56) and begins echolocating (11:23:58) at depth with dolphins intermittently observed in low light conditions near the whale's head. Corresponding depth and activity profiles including a successful prey capture recorded during filming are shown in Fig. 7.
